# An overview of fermentation in the food industry - looking back from a new perspective

**DOI:** 10.1186/s40643-023-00702-y

**Published:** 2023-11-28

**Authors:** Shahida Anusha Siddiqui, Zeki Erol, Jerina Rugji, Fulya Taşçı, Hatice Ahu Kahraman, Valeria Toppi, Laura Musa, Giacomo Di Giacinto, Nur Alim Bahmid, Mohammad Mehdizadeh, Roberto Castro-Muñoz

**Affiliations:** 1 Technical University of Munich, Campus Straubing for Biotechnology and Sustainability, Essigberg 3, 94315 Straubing, Germany; 2 German Institute of Food Technologies (DIL E.V.), Prof.-Von-Klitzing Str. 7, 49610 Quakenbrück, Germany; 3 Department of Food Hygiene and Technology, Faculty of Veterinary Medicine, Burdur Mehmet Akif Ersoy University, İstiklal Campus, 15030 Burdur, Turkey; 4 Department of Veterinary Medicine, University of Perugia, 06126 Perugia, Italy; 5 Department of Veterinary Medicine and Animal Sciences, University of Milan, 26900 Lodi, Italy; 6 Research Center for Food Technology and Processing, National Research and Innovation Agency (BRIN), Gading, Playen, Gunungkidul, 55861 Yogyakarta, Indonesia; 7 Faculty of Agriculture and Natural Resources, University of Mohaghegh Ardabili, Ardabil, Iran; 8 Ilam Science and Technology Park, Ilam, Iran; 9 Tecnologico de Monterrey, Campus Toluca, Av. Eduardo Monroy Cárdenas 2000, San Antonio Buenavista, 50110 Toluca de Lerdo, Mexico; 10 Department of Sanitary Engineering, Faculty of Civil and Environmental Engineering, Gdansk University of Technology, G. Narutowicza St. 11/12, 80-233 Gdansk, Poland

**Keywords:** Fermentation, Non-communicable disease, Waste valorization, Fermented dairy, Fermented meat, Fermented plant-based foods

## Abstract

Fermentation is thought to be born in the Fertile Crescent, and since then, almost every culture has integrated fermented foods into their dietary habits. Originally used to preserve foods, fermentation is now applied to improve their physicochemical, sensory, nutritional, and safety attributes. Fermented dairy, alcoholic beverages like wine and beer, fermented vegetables, fruits, and meats are all highly valuable due to their increased storage stability, reduced risk of food poisoning, and enhanced flavor. Over the years, scientific research has associated the consumption of fermented products with improved health status. The fermentation process helps to break down compounds into more easily digestible forms. It also helps to reduce the amount of toxins and pathogens in food. Additionally, fermented foods contain probiotics, which are beneficial bacteria that help the body to digest food and absorb nutrients. In today’s world, non-communicable diseases such as cardiovascular disease, type 2 diabetes, cancer, and allergies have increased. In this regard, scientific investigations have demonstrated that shifting to a diet that contains fermented foods can reduce the risk of non-communicable diseases. Moreover, in the last decade, there has been a growing interest in fermentation technology to valorize food waste into valuable by-products. Fermentation of various food wastes has resulted in the successful production of valuable by-products, including enzymes, pigments, and biofuels.

## Introduction

The growing concern about the availability of safe and sustainable natural food supplies is a direct result of a rapidly expanding global population (Knorr and Augustin 2022). For the production of the next generation of food components and products, fermentation stands out among other approaches like cellular or acellular products, edible biomass, and edible insects. Compared to conventional farming, the production of fermented products requires reduced land, generates fewer greenhouse gas emissions, and consumes less water. Additionally, fermented foods have been linked with health benefits such as lowering cholesterol levels, boosting the immune system, protecting against infections, cancer, osteoporosis, obesity, diabetes, allergies, atherosclerosis, and reducing lactose sensitivity (Tamang and Thapa 2022). The beneficial effects of fermented foods are mostly credited to bioactive peptide fractions produced during fermentation through microbial protein breakdown (Şanlier et al. 2019).

Fermentation is a time-honored method used for ages to prolong the shelf life of food and augment its nutritional quality (Augustin et al. 2023). It has been used to preserve various animal-derived, seafood, and plant-originated foods. Middle Eastern, European, and Indian cultures developed cheese, cultured milk, and fermentation-based milk products. On the contrary, animal husbandry was more restricted to Eastern countries including Japan, Korea, and China. A wide range of ingredients used to produce alcoholic beverages and fermented foods was also influenced by cultural, social, religious, and economic variables. Therefore, fermented foods that originated in Asia tended to be based on rice and grains, vegetables, fish, and soybeans, while in Africa, native cereal grains such as millet, sorghum, maize, and wheat represent popular fermenting substrates (Tamang et al. 2020). The regulated action of microorganisms on a food substrate is at the root of fermentation. Fermentation may be spontaneously induced by microorganisms commonly found in raw ingredients. Traditional sauerkraut, sourdough bread, and kimchi result from fermented foods rendered through spontaneous fermentation. Advances in biotechnology have made fermentation into an established process. Using starting cultures allow for acquiring a standardized food product (Bourdichon et al. 2021). For food applications, strain engineering often involves harmless microorganisms like *Bacillus* spp., yeast like *Saccharomyces cerevisiae*, *Komagataella phaffii*, *Kluyveromyces* spp., or filamentous fungi (Augustin et al. 2023; Chai et al. 2022a, b; Vieira Gomes et al. 2018). Specific food components like antioxidants, colorants, flavors, enzymes, and vitamins can also be manufactured through fermentation (Santiago‐Díaz et al. 2022). Antioxidants, bacteriocins, pigments, enzymes, and other food components are increasingly being manufactured utilizing food waste as a fermentation substrate. A direct connection exists between eliminating food waste and developing creative, high-value assets generated by applying an under- or untapped resource (Yang et al. 2022). In the bargain, scientific investigations have ensured insights into using fermentation technology to produce fermented edible insects to promote food security. There are already available fermented insect-containing edible preparations such as sauces, powder, paste, and fermented dishes containing insects with the possibility of extension (Kewuyemi et al. 2020). Examples of such fermented sauces include those made using *Locusta migratoria* and *Galleria mellonella*. These sauces were very satisfactory compared with conventional seafood sauces (Mouritsen et al. 2017).

Fermentation further improves compound extraction efficiency, alters the antioxidant profile, and generates novel bioactive compounds from the food matrix. Lately, there is an emerging understanding that precision fermentation represents a key innovation in the food industry’s impending fourth industrial revolution. Modernizing food production with precision fermentation is becoming increasingly popular. Because of its potential to reduce waste and increase the efficiency of protein, lipid, and carbohydrate production (Augustin et al. 2023). This overview will discuss fermentation’s cultural significance and ecological importance, technological advancements of fermented beverage and food worldwide, health benefits, nutritional value, and microbiological insights into various fermented foods. We will examine distinct types of fermented foods of animal, plant, microalgae, and edible insect origin. For every kind of product, bacteria involved in fermentation, consumers’ acceptance, place of origin, and consumption will be discussed. There is also a discussion of how the industrial revolution has impacted the process of fermented foods and how fermentation is used to value food waste.

## Technology advancements in fermentation

The process of fermentation encompasses the biochemical activity of organisms throughout their life cycle, from growth to death. Industrial-scale production of food, pharmaceuticals, and alcoholic beverages utilizes fermentation technology powered by these organisms. Industrial fermentation technology relies on the fundamental principle of cultivating organisms in optimal conditions. This is achieved by providing the required raw materials, including carbon, nitrogen, salts, trace elements, and vitamins, essential for their growth (Sharma et al. 2020). Additionally, the temperature, pH, and oxygen concentrations must be controlled in order to ensure the highest possible yield of the desired product. The fermentation process is also closely monitored to ensure that the organisms are not exposed to any toxins or pathogens that could potentially affect the outcome. The mounting focus on environmental conservation and renewable energy has sparked a surge of interest in the retrieval of fermentation products, including industrial chemicals, organic acids, and feed or food additives. This upswing has caused a proliferation of products beyond the customary high-value, low-volume pharmaceuticals, with fermentation now rivaling the synthesis of commodity chemicals itself. Companies must optimize efficiency and reduce waste by-products to remain competitive in producing low-cost, high-volume chemicals. Currently, scientific communities are keenly exploring the biotechnological potential of agro-industrial remnants.

Emerging fermentation-based technologies have revolutionized the food industry in various ways. Cai et al. (2020) reported that sulforaphane yields were increased by 16 times when broccoli florets were pre-heated at 65 °C for 3 min followed by maceration and lactic acid bacteria fermentation in a laboratory scale. Additionally, these technologies have paved the way for producing a pea protein hydrolysate component that enhances the saltiness of food. In a study by Xu et al. (2020), fermentation of carrot juice was found to enhance the nutritional profile of carrot juice by using probiotic *Lactobacillus gasseri*. Fermented straight carrot juice was found to have a reduction in sugar (27%) according to their results. It is possible to improve fermentation processes and tweak fermented foods’ nutritional and sensory attributes using these methods. To enhance fermentation, the following factors at each stage need to be addressed: selecting and designing of targets, strain optimization, bioprocess development, feedstock improvement, and final product formulation and production. One fermentation technique utilized by various industries, including food, pharmaceuticals, and textiles, is solid-state fermentation (SSF), which involves utilizing solid support rather than a liquid to produce microorganism metabolites. SSF boasts several benefits: minimal waste production and a reduced environmental impact, natural solids as a medium with low energy costs and capital investments, no sterilization requirement, improved downstream processes and reduced microbial contaminants (Sun et al. 2022).

Acidogenic fermentation (AF) is crucial in producing valuable chemicals like C1–C6 carboxylic acids and alcohol. However, low product titers have been a persistent challenge due to thermodynamic limitations. Recent research shows that boosting the redox potential in AF can enhance metabolic pathways, enabling a smoother flow of electrons and lowering activation energy barriers. This improves substrate utilization rates, product yields, and speciation. This augmented system, known as electro-fermentation (EF), has tremendous potential to revolutionize fermentation technology by offering an exogenous electricity supply (Chandrasekhar and Venkata Mohan 2014; Luo et al. 2022; Nagarajan et al. 2022).

Ultrasound waves that exceed 20 kHz have emerged as an eco-friendly option for processing agri-foods. This sound technology applies a cavitation process, during the formation of bubbles and their burst, leading to a sterilization effect on food and drink products. Ultrasonication aids in the deactivation of enzymes and microorganisms by disrupting the cell membrane (Gavahian et al. 2022). Ultrasonic systems are easily applicable on an industrial scale as they do not require immersion of the product into a liquid medium. This allows hydrophilic nutritional compounds to be maintained, enabling these systems to be employed on a large scale. Wineries have successfully employed this emerging technology to enhance wine aroma, flavor, color, and phenolic profile. Research has uncovered the benefits of ultrasonic technology on wine fermentation and aging. This innovative approach improves wine quality by increasing the key aging indicators including phenolic substances and color intensities and deactivates microbes. Furthermore, ultrasound application results in enhanced physiological, phytochemical, biochemical, and organoleptic characteristics of alcoholic drinks. Celotti et al. (2020) investigated the influence of high power ultrasound on anthocyanins and phenolic levels in red young wines. Following 15 and 30 days of storage, the tannin content of the treated wine decreased by 15% and 40%, respectively, due to the higher ultrasound amplitude (81%). This suggests that high power ultrasound can be used to significantly reduce the tannin content of red young wines, making them less bitter and more palatable. Moreover, Zhang et al. (2016) studied the physicochemical characteristics of red wine after ultrasound treatment. 240 W of power ultrasound, a frequency of 80 kHz, and a temperature of 20 °C are considered optimal conditions for ultrasound application in red wine processing. A significant change was observed in total phenolic compounds, electrical conductivity, and chromatic characteristics of the samples. However, pH or acidity titratable did not differ significantly. These studies provide evidence that ultrasound treatment can be used as an effective tool to manipulate the physicochemical characteristics of red wine.

The benefits of producing lactic acid from renewable sources have sparked much interest across different fields. The petrochemical industry has embraced this approach for its high yield and cost-effective productivity using readily available substrates (Zhao et al. 2016). However, the disposal of biomass and waste materials from various sources poses a significant environmental challenge (RedCorn and Engelberth 2016). The ideal solution is an integrated biorefinery platform that produces high-value bioproducts while addressing waste management. The potential applications of fermentation-produced optical pure lactic acid in the food, pharmaceutical, cosmetic, and the textile industry have made it a highly promising option for packaging materials. Polylactic acid, derived from natural resources, is a green substitute for petrochemical-based bioplastics. While biodegradability is a significant advantage, lactic acid's high price production has hindered the widespread application of this material (Bastidas-Oyanedel et al. 2015; Kumar et al. 2019).

In developing countries, small-scale food fermentation technologies have been refined through practical wisdom rather than scientific innovation. As a result, many manufacturers need more certainty about modernizing and altering their fermentation processes. However, it is crucial to enhance the safety and quality of fermented foods while preserving their distinctiveness and keeping production costs low. One practical approach is the consortium method, which has successfully improved Thailand's small-scale soy sauce fermentation (O’toole 2019). This approach facilitates ongoing collaboration between industry and scientists, providing the latter with the necessary research focus to help the industry thrive. While the small-scale fermentation industry has hesitated to embrace starter cultures due to concerns over the loss of unique flavor, modern molecular biology techniques have ushered in a new era of tailored starter culture development. In recent studies, microflora from distinct product origins exhibited variations in sensory quality and composition, highlighting the potential for customization. To support this trend, a cell bank is currently in the works as a resource for Thai fermented pork sausage, intending to ease the implementation of starter cultures in production (Østergaard et al. 1998; Mongkolwai et al. 1997; Valyasevi and Rolle 2002).

## Fermentation in the food industry as an environmentally friendly alternative to improve nutritional value

Global population growth often correlates with increased demand for food and energy. Large amounts of food waste have been generated by industrialization and lack of proper waste management strategies (Ng et al. 2020). By 2030, 2.1 billion tons of food waste will be generated annually, according to a Boston Consulting Group report (Martin-Rios et al. 2021). The agricultural and food processing industries face two major challenges. On the one hand, it is critical to limit their impact on the environment to minimize climate change effects. Conversely, the insufficiency of superior alternatives to health-promoting diets (Rastogi et al. 2022). Food shortages and environmental consequences result from lost and wasted agricultural output during processing and distribution (Read et al. 2020). A high proportion of nutrients are found in plant-based food processing waste, including pulp, peels, and silage, but these materials are typically disposed of in landfills or washed into water bodies, causing the fast depletion of dissolved oxygen (Ishangulyyev et al. 2019). A high sugar, refined carb, processed meat, artificial additive, and trans-fat diet is also linked to obesity, Type 2 diabetes (T2D), hormonal imbalances, and cardiovascular disease (Tandon et al. 2022). Food fermentation technology has a number of advantages, including environmental and health benefits (Paramithiotis et al. 2022; Rastogi et al. 2022). In fermented foods, microorganisms and enzymes are involved in the enzymatic transformation of food substances, emphasizing microbial changes as the distinguishing feature.

**Fig. 1:**
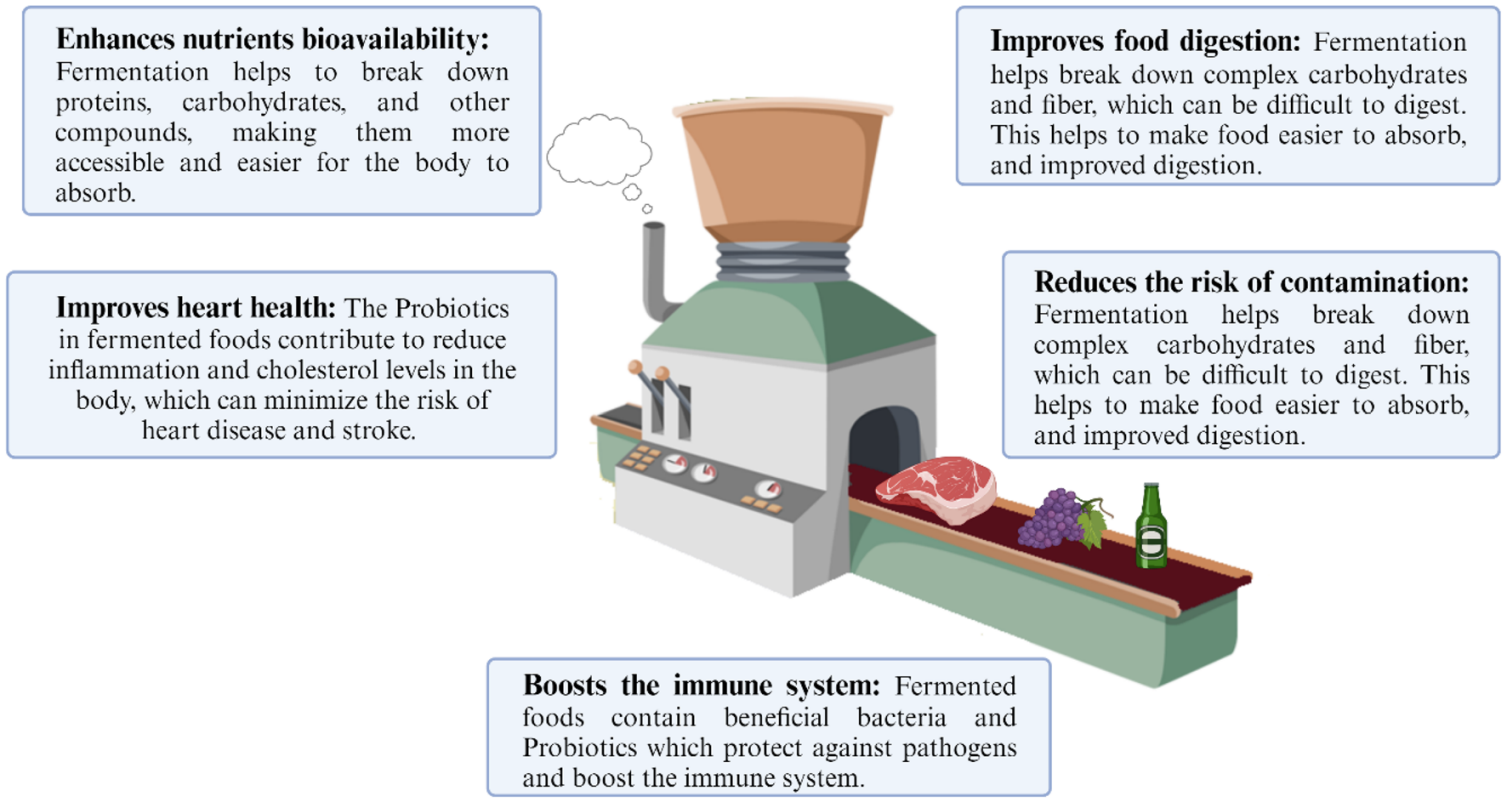
Fermentation’s primary benefits. “Created with BioRender.com”

A wide range of fermented products make up FFs, including fermented meat, fish, dairy products, fermented fruits, and alcoholic drinks. Furthermore, they include vinegar, cocoa, soy sauce, fish sauce, and coffee (Gänzle 2022). Industrial FF production has evolved from household techniques. Fermented beverages are uncontrolled at household levels due to ubiquitous microorganisms. Traditional fermentation, despite being economical, also preserves food (Adesulu and Awojobi 2014). A functional microorganism provides the health benefits of antibacterial, antioxidant, and peptide synthesis in FFs. Additionally, fermentation can produce nutritious foods and sustainable food supplies. Compared to traditional chemical synthesis methods, fermentation is more flexible, cost-effective, and environmentally friendly (Fig. 1). There has been more investigation into fermentation-based synthesis, optimization, and downstream of medicines, biofuels, biofertilizers, and biodegradable polymers (Rastogi et al. 2022). Microbiological fermentation converts green waste into valuable products. Food waste is considered edible or inedible fractions originating from animals or plants generated before or after consumption (Torres-León et al. 2021). Fermentation technique for bioconversion is significantly influenced by the type of food waste used. Solid-state fermentation is often performed on solid substrates to improve nutrient efficiency, while submerged fermentation is generally used on liquid substrates (Sadh et al. 2018). Due to its low cost, high productivity, and simplicity, submerged fermentation is more commonly used in industrial-scale fermentation. Food waste is considered an excellent input for microbial fermentation due to its abundance of phenolics, proteins, fatty acids, minerals, and several bioactive ingredients. Therefore, the biotransformation of these rich sources allows value-added outputs without waste treatment (Dursun and Dalgıç, 2016; Ng et al. 2020). Food processing wastes can be used to produce enzymes, biofuels, oligosaccharides, growth-promoting agents, polysaccharides, bioplastics, proteins, and bioactive compounds (Sadh et al. 2018; Torres-León et al. 2021).

Lipids extracted from waste materials can also be precursors to bioethanol synthesis (96%) through fermentation (Ashokkumar et al. 2022). Reducing waste, increasing energy production, and developing other healthcare products are all enhanced by transforming organic waste into value-added bioproducts. Bioproducts such as protease enzyme have been produced by microbial fermentation of food waste (De Castro et al. 2015; Mathias et al. 2017), β-glucosidase enzyme using brewer´s spent grain (BSG) as substrate (Leite et al. 2019), antioxidant peptides using microorganism fermentation (*Aspergillus oryzae*) and turbot skin as substrate (Fang et al. 2017). Sepúlveda et al. (2020) conducted a study to develop ellagic acid using polyphenols from orange peel wastes through submerged fermentation. This study demonstrated the effectiveness of this method for converting molecules present in orange waste to produce high-value products like ellagic acid. A combined submerged and solid-state fermentation method using *Neurospora intermedia* was successfully used by Gmoser et al. (2019) for the conversion of waste bread into protein and carotenoids. By using wheat waste as a substrate, Dursun and Dalgıç (2016) achieved astaxanthin pigment production by four different yeast species. It is difficult to estimate the bioproducts produced through bioconversion processes due to the unidentified exact amounts of bioactive content of food waste. Thus, innovative biotechnology techniques must be employed to optimize the reutilization and recycling of food-processing and agricultural wastes (Ng et al. 2020).

Consumers' desire for healthier foods drives manufacturers to seek out new methods of preparing food (Adebo et al. 2018). While FFs were initially developed to prolong foods’ shelf-life, they are now commonly used to enhance food safety, sensory quality, and nutritional value (Macori and Cotter 2018). Lately, there has been a surge in awareness of FFs as possible sources of functional foods (Adebo et al. 2018). The fermentation process alters substrates and generates biologically active or biologically available final products, improving nutritional and functional properties. Fermentation by-products such as organic acids, ethanol, and bacteriocins minimize contamination risk (Marco et al. 2017). Beneficial microorganisms, or derivatives obtained from fermentation may contribute to the health benefits of FF consumption (Adebo et al. 2018; Macori and Cotter 2018; Marco et al. 2017).

Fermentation changes the nutritional and bioactive qualities of food matrices. This is due to the combined effects of the raw material's enzymatic activity and microorganism metabolism (Savaiano 2014). In plant-based fermented foods, lactic acid bacteria produce glycosyl hydrolase, esterase, decarboxylase, and phenolic acid reductase to convert phenolic substances into physiologically active metabolites (Filannino et al. 2015). The fermented dairy products contain biologically active peptides such as yogurt, cheese, fermented milk, kefir, dahi, etc. These peptides have anti-thrombotic, anti-hypertensive, immune-modulatory, osteogenic, and antioxidant properties. LABs generate biological peptides by breaking down dairy proteins (Pihlanto and Korhonen 2015). Fermentation metabolites vary by strain, but lactic acid (LA) is the primary product. Lactic acid can reach 1% in several LAB fermentations. Pro-inflammatory cytokines, bone marrow-derived macrophages, and dendritic cells are diminished by LA in a dose-dependent manner (Iraporda et al. 2015; Marco et al. 2017). Despite the enhanced availability of trace minerals and vitamins, fermentation of plant and dairy matrices amplifies the production of vitamins, particularly folate, riboflavin, and B12 (Macori and Cotter 2018; Marco et al. 2017). Fermentation quality depends strongly on the activity of the microorganisms involved in the process and the substrate used in fermentation. As prebiotics, fibers enhance fermentation bacteria's microbial population, while also influencing the biochemical profile of the final products with health benefits (Adebo et al. 2018). Intestinal health can be positively impacted by short-chain fatty acids including butyric acid, propionic acid, and acetic acid derived from dietary fiber fermentation (Hati et al. 2019; Kang et al. 2021). Fermentates may also provide some health benefits of FFs. A fermentate is a powdered preparation incorporating beneficial bacteria, metabolites, and bioactive components derived from fermentation and substrates (Mathur et al. 2020). A range of fermentates containing different ingredients have been shown to have beneficial outcomes, including effects on gut health due to the production of lactose-hydrolyzed products for lactose intolerant individuals and the production of glycosylated products using *β*-galactosidases enzyme (Saqib et al. 2017), regulates food intake and prevents weight loss using glucagon-like peptide-1 (GLP-1) (Cho et al. 2014; van Bloemendaal et al. 2014), the anti-inflammatory activity of paraprobiotic CP2305 (*Lactobacillus gasseri* CP2305) by affecting the growth of fecal* Bacteroides vulgatus* and *Dorea longicatena*, which are involved in intestinal infection (Nishida et al. 2017), as well as the potential benefits of heat-killed bacteria in treating dermatological disorders (Piqué et al. 2019). Heat-killed Lactobacillus kunkeei YB38 increased bowel movements and improved the intestinal environment in humans, according to Asama et al. (2016). Warda et al. (2019) found that long-term intake of an ADR-159 diet containing inactivated lactobacilli had no negative effects on health outcomes of male mice.

### Fermented foods and non-communicable diseases

Dietary habits are significant in the hierarchy of essential factors triggering non-communicable diseases (NCD). High saturated fatty acids, sodium, a sedentary lifestyle, and a diet poor in fruits and vegetables are some risk factors for developing NCD (Angeles-Agdeppa et al. 2020). From a health stance, integrating FFs into the daily diet has been in the spotlight of several investigations. The presence of beneficial microorganisms such as LABs concomitantly with their biologically active metabolites has been associated with several positive effects concerning NCD (Mathur et al. 2020). LAB fermentation technologies provide various bioactive substances with potential health benefits (Mathur et al. 2020).

The health benefits generated from fermented products are mainly regarded as the metabolic activities of the fermenting microbial community or their biologically active metabolites (Table 1). As an example, various LABs have been found to produce exopolysaccharides (EPSs) during fermentation. The produced EPSs have been related to different health benefits, including antidiabetic, cholesterol-reducing, antioxidant, and immunomodulatory effects (Nampoothiri et al. 2017; Patel and Prajapat 2013). EPSs derived from LABs exhibit their anti-cholesterol activity by alleviating cholesterol assimilation in the intestine and inducing the release of bile acids (Nampoothiri et al. 2017). Among FFs, fermented dairy products have been linked to a variety of health benefits. Dairy products represent a rich source of proteins, fat, and lactose which generate different bioactive molecules due to their enzymatic activities. During fermentation, several different LAB strains may secrete enzymes that help break down complicated inedible substrates into more easily digestible ones. These enzymes include β-galactosidase, amylase, protease, lipase, and glucoamylase (Macori and Cotter 2018; Marco et al. 2017). The secretion of the β-galactosidase enzyme facilitates the breakdown of lactose, thus addressing lactose intolerance. Despite facilitating nutrient absorption, particularly in fermented cereals, LABs contribute to removing toxic compounds such as phytic acid by enhancing phytase activity (Marco et al. 2017).

**Table 1:** Fermented products and health relationship

Category	Food matrix	Microorganisms	Influential compound	Health benefits	References
Fermented dairy	Milk	*Lactobacillus plantarum* PU11	ACE-inhibitory peptides and GABA	Anti-hypertensive	Nejati et al. (2013)
Fermented dairy	Milk	*L. lactis* NRRL B-50 571, *L. lactis* NRRL B-50 572	ACE-inhibitory peptides	Hypotensive and heart rate-lowering	Rodríguez-Figueroa et al. (2013)
Fermented dairy	Kefir	Mixed flora	Not specified	Antifungal effect	Gamba et al. (2016)
Fermented plant-based foods	Dosa batter, curd, handva batter, soycurd and fermented cabbage	*L. fermentum* PH5, PD2	Not specified	Hypocholesterolemic effect	Thakkar et al. (2020)
Fermented dairy	Kule naoto	*L. rhamnosus* BFE5264 and *L. plantarum* NR74	Not specified	Reduction in cholesterol absorption in Caco-2 cells	Yoon et al. (2013)
Fermented edible insects	Mealworm and Grasshopper flour	*L. lactis* (NRRL B-50571, NRRL B-50572	Polyphenolic compounds	Antioxidant and antihypertensive activity	Mendoza-Salazar et al. (2021)
Fermented plant-derived milk	Soy milk	*L. rhamnosus* C2, C6 and *L. casei* NCDC 17, 297	Polyphenolic compounds	Antioxidative activity	Subrota et al. (2013)
Fermented dairy	Fermented goat milk with passion fruit by-product	*Lactobacillus casei* Lc-1, *Streptococcus thermophilus* TA040	Short chain fatty acids, polyphenolic compounds	Modulation of gut microbiota	Neves Casarotti et al. (2020)
Fermented plant-based foods	Pag-sian-dorng	*L. plantarum* BCC 47723	Complex glycopolymers and proteins, teichoic acids found in cell surface	Zearalenone removal	Adunphatcharaphon et al. (2021)
Fermented dairy	Cow, Buffalo and goat milk	*Lactobacillus helveticus, Lactobacillus delbrueckii* subsp*. bulgaricus,* and* Streptococcus thermophilus*	Proteolytic enzymes	Casein allergy	Anggraini et al. (2018)
Fermented dairy	Bovine milk	*Enterococcus faecalis* VB63F	Proteolytic enzymes	Hypoallergenic	Biscola et al. (2016)
Fermented plant-based foods	Peanut pulp	*Bacillus natto*	Proteolytic enzymes	Hypoallergenic	Jiang et al. (2020)
Fermented plant-based foods	Soybeans	*Bacillus subtilis*	Proteolytic enzymes	Hypoallergenic	Yang et al. (2020)
Fermented plant-based foods	*Myriciaria dubia* Mc. Vaugh	*L. helveticus (*ATCC 12046*)* and *L. plantarum (*NCDO 1193*)*	α-Amylaseα-Glucosidase	Anti-diabetes, hypertension	Fujita et al. (2017)
Fermented plant-based foods	Moringa oleifera	LAB	Not specified	Decreases hepatic adiposity and ameliorates Glucose intolerance	Joung et al. (2017)
Fermented plant-based foods	*Carica papaya,* L	Not specified	Papain	protection from the oxidative damage associated with diabetes	Raffaelli et al. (2015)

Intake of fermented dairy was correlated with a lower incidence of cardiovascular diseases. Furthermore, additional research quantifying the functional impact and side effects may give a more understandable mechanism (Zhang et al. 2020a). Moreover, anti-hypertensive angiotensin-converting-enzyme (ACE) inhibitor peptides in fermented dairy products are particularly interesting (Fekete et al. 2015). The anti-hypertensive effect is also induced by several bioactive compounds in fermented dairy, including valyl-prolyl-proline and isoleucyl-prolyl-proline (Nongonierma and FitzGerald 2015). Among NCDs, cancer has been regarded as one of the most critical diseases closely related to dietary habits. It is predicted that 30–40% of cancer cases may be averted by modifying risk factors and behavioral modifications, the most important of which is food. There is evidence that suggests that consuming FFs may lower cancer risk due to the presence of specific nutrients (Tasdemir and Sanlier 2020). The occurrence of diverse reactive oxygen species and other free radicals due to various metabolic reactions beyond the tolerance levels disrupts body homeostasis. The presence of disorders at the cellular level advances the appearance of cancer. Antioxidants are assumed to impair carcinogenesis by preventing DNA from free radicals, slowing cell proliferation in response to oxidants, and triggering apoptosis (Khurana et al. 2018; Tasdemir and Sanlier 2020). Fermentation is finalized by producing specific products with robust antioxidant properties, including phenolic compounds, anthocyanins, EPSs, and bioactive peptides (Kok and Hutkins 2018; Marco et al. 2017; Tasdemir and Sanlier 2020). Gao et al. (2013) have outlined the anti-tumor effects of bioactive substances generated from FFs on the gastric cancer cell line SGC7901. Conversely, T2D significantly contributes to an individual’s reduced life expectancy (Sivamaruthi et al. 2018). Several in vitro, in vivo, and clinical investigations have reported the antidiabetic impacts of FFs. (Fujita et al. 2017) have noted that *camu-camu* fermented with LABs can support T2D and hypertension. Furthermore, Raffaelli et al. (2015) outlined that fermented papaya fruit can mitigate diabetes-related oxidative damage. By increasing membrane permeability, fermented papaya fruit improves platelet function. Fermented *Moringa oleifera* could ameliorate glucose intolerance in high-fat diet obese mice (Joung et al. 2017).

Fermented foods have been reported to have an antidiabetic effect in numerous scientific studies. Algonaiman et al. (2022) investigated the antidiabetic and hypolipidemic properties of fermented oat extract in rats. The study found that the fermented oat extract had a significant effect on blood glucose levels and lipid profile of the rats. It also improved body weight and decreased oxidative stress in the rats. The results suggest that fermented oat extract may be a potential alternative for the management of diabetes. The antidiabetic properties of lactic acid bacteria isolated from traditional fermented foods were evaluated by Cai et al. (2019). The results showed that the lactic acid bacteria were effective in reducing blood glucose levels and had a protective effect against oxidative stress. This suggests that lactic acid bacteria could be used to develop functional food products for the management of diabetes. Two Lactobacillus species were used in the study by Feng et al. (2018a, b) to assess fermented buckwheat's antidiabetic effects. They reported that tartary buckwheat fermented with *L. plantarum* TK9 and *L. paracasei* TK1501 has the potential to regulate blood glucose in diabetics. Antidiabetic effects of fermented lettuce extracts were studied by Jeong et al. (2021). This study indicates fermented lettuce extract could be considered an effective additive to diabetic foods to balance blood glucose levels and improve insulin resistance. The antibacterial and antidiabetic properties of synbiotic fermented milk have been studied by Shafi et al. (2019). In diabetic rabbits, the product was found to reduce blood glucose levels, urea levels, and creatinine levels. Antioxidant, antimicrobial, and antidiabetic activities of probiotic-fermented blueberry juices were significantly improved compared to non-fermented juices (Zhong et al. 2021).

Additionally, bioactive metabolites of fermentation are well-known for their immune-modulatory properties. Consumption of FFs has been associated with increased IgA-producing cells and enhanced macrophage activity (Park and Bae 2016). Immunomodulation is related to the generation of cytokines such as IL-12, tumor necrosis factor-gamma (TNF-y), and interferon-y (IFN-y) as a consequence of increased T cell and dendritic cell activity (Jones et al. 2014). Irritable bowel syndrome (IBS), for example, may also be controlled with fermented foods like kimchi, Crohn’s disease, and infections caused by external invasions or poor eating habits (Han et al. 2020; Kim and Park 2018; Seong et al. 2021). Glucagon-like peptide-1 (GLP-1) is a significant hormone linked to suppressed appetite, and it has been hypothesized that inducing GLP-1 reduces appetite and food consumption (Cho et al. 2014; van Bloemendaal et al. 2014). (Chaudhari et al. 2017) have reported that fermentates of *Lactobacillus helveticus* induce the proglucagon and secretion of GLP-1 in STC-1 (pGIP/Neo) cells.

### Food allergies

Allergy is one of the most severe NCD associated with food intake. Although food-related allergies are estimated to affect 1–10% of the population, the prevalence of the condition is reported to be higher in children, reaching 5–12.6% (Pi et al. 2022). Foods like milk, egg, shellfish, peanut, soy, wheat, and fish are most commonly associated with allergies (Ricci et al. 2019). Food allergies typically appear within two hours of exposure because there is no immunological and clinical tolerance to the food allergen (Barni et al. 2020). Because it is almost impossible to exclude allergens from dietary patterns, processing these items has received greater significance (Fu et al. 2019). Physical, chemical, and biological processes may reduce allergens in food. Compared to other existing procedures, fermentation is also convenient and safe, besides improving the physicochemical and nutritional value of the foods (Pi et al. 2019). Fermentation alleviates food allergies in several ways. High molecular substances, including proteins, may be broken down through fermentation into more tolerable low molecular entities like small molecule polypeptides and amino acids, causing an alteration in structure and altered characteristics (Fu et al. 2019).

A number of pathways occur during fermentation that reduce the allergenicity of food allergens, such as proteolytic enzymes, denaturation by acidic environments, glycosylation, and the Maillard reaction (Pi et al. 2019). Several metabolites formed from fermentation can regulate the immune system by adjusting the proliferation and cytokine release from T cells, Th 17 cells, and Treg cells (Dargahi et al. 2019). For instance, propionic acid produced from fermentation induces dendritic cells to generate TGF-b and retinoic acid. TGF-b and retinoic acid cause a switch of naive T cells to Tregs and inhibit Th2 generation. Additionally, fermentation metabolites such as 10-hydroxy cis-12-octadecenoic acid and linoleic acid escalate intestinal barrier function and lower systemic food allergen levels (Hirata and Kunisawa 2017). Moreover, the soy-derived acidic polysaccharide APS-I has also been shown to stimulate IgA synthesis in the intestines (Cao et al. 2019). pH, temperature, and substrate are among the processing parameters in the allergenicity reduction is microbial strain utilized in fermentation. Fermentation's influence on dietary allergies is most visible in some foods, such as peanuts, soybeans, milk, etc. (Fu et al. 2019). For instance, allergenicity in fermented cow, buffalo, and goat milk was reduced after co-cultivation of *Lactobacillus helveticus*, *Lactobacillus delbrueckii* subsp. *bulgaricus*, and *Streptococcus thermophilus* (Anggraini et al. 2018). Likewise, the proteolytic activity of *Enterococcus faecalis* VB63F decreased the allergenicity of fermented bovine milk by degrading the protein fraction (Bos d 9–11) responsible for the allergic reaction (Biscola et al. 2016). *Bacillus* spp. has been reported to exhibit anti-allergic effects when used in several plant-based foods' fermentation. For instance, results showed that the allergenicity of peanut protein could be reduced by using *Bacillus natto* in a fermentation process using peanut pulp (Jiang et al. 2020). Moreover, according to Yang et al. (2020), *Bacillus subtilis* showed the most significant hypoallergenic impact (44.5%) in the allergenicity of soybean when compared to other fermenting bacteria. Mecherfi et al. (2019) reported that *Lactococcus lactis* reduced gluten-related sensitization. The primary epitopes responsible for wheat allergy were found in the gliadin repeating domain hydrolyzed by *L. lactis*. Multiple bacterial strains used in fermentation are more effective in alleviating food allergenicity. Nevertheless, it should not be underestimated that some strains can produce bitter peptides that negatively affect the taste of the fermented product. Consequently, fermenting food offers a new approach to lowering the allergenicity of diverse foods when combined with other processing methods, such as heat treatment, pulsed light, and ultrasonication (Pi et al. 2019).

## Fermentation microbiology

Fermentation is an ancient activity dating back to the earliest human civilizations when scientific principles were not yet recognized and studied (El-Mansi et al. 2019). According to the International Scientific Association for Probiotics and Prebiotics (ISSAP), foods that have undergone microbial growth and enzymatic conversion are defined as fermented foods (Marco et al. 2021). Fermentation helps to preserve foods, making them last longer and making them easier to digest. It also adds flavor, texture, and nutrition to foods, making them more appealing to consumers. Additionally, fermentation helps to break down complex compounds into simpler forms that can be more easily absorbed by the body. During food fermentation, complex components are broken down into simpler parts, many of which may be biologically active, using microorganisms (Macori and Cotter 2018). Available scientific data indicate that many fermented foods contain both nutritive and non-nutritional components that can control particular target processes in vivo related to consumer well-being and health (Tamang et al. 2016). The presence of microorganisms in fermented foods can provide consumers with numerous health benefits, such as probiotic properties, antibacterial properties, antioxidant properties, and peptide synthesis (Tamang et al. 2016). Therefore, regular consumption of fermented foods may significantly contribute to improved human health.

Fermentation is a widely used process in the food industry, and its products are diverse and beneficial to our health. Different types of fermentation can be categorized based on the microorganisms involved in the fermentation process, the primary metabolites produced by these microorganisms, the raw materials used, the fermentation method, oxygen demand, pH level, and nutritional metabolism (Vilela et al. 2019). The most common category, based on the microorganisms responsible for the fermentation and its product, is acetic acid (vinegar), lactic acid (dairy products, vegetables, cereals, meat), ethanol/alcohol (baking, brewing, winemaking), alkaline (Japanese natto) (Vilela et al. 2019). Fermentation typically involves the utilization of compounds from the feedstock by microorganisms such as yeast and lactic acid bacteria (LAB) like Lactobacillus, Lactococcus, Pediococcus, Enterococcus, and Streptococcus, commonly found in fermented milk products. These microorganisms produce a variety of compounds, including alcohols, acids, and gases, during the fermentation process. These compounds can be used to produce a variety of products, such as fermented dairy products and alcoholic beverages. As a subfield of bioengineering, fermentation has gained increasing attention from a variety of fields, including microbiology, chemical engineering, genetic engineering, cell engineering, mechanical engineering, software, and computer hardware (Feng et al. 2018b). There is no doubt that fermentation is a significant field of research and that its potential applications are still expanding. Fermentation can be applied to manufacture pharmaceuticals, chemicals, and biofuels, and produce food, beverages, and animal feed. It is an efficient and cost-effective process, and can also be used to produce renewable energy. Multiple microbe interactions lead to fermented foods, primarily traditional fermented foods. During food fermentation, flavor-active compounds form due to proteolysis and hydrolysis of peptides, with the conversion of amino acids and the formation of flavor-enhancing amino acid compounds (Zhao et al. 2016). Many fermented filtrates or extracts have potential health benefits, including high nutritional value, antioxidant activity, gut microbiome balance, and immune enhancement (Kim et al. 2019). Highly selective biocatalytic processes regulate complex systems of biochemical reactions. It should be noted, however, that not all enzymes are 100% selective. Proteases, for instance, can freely bind to many substrates with similar chemical structures, reducing their efficiency. Thus, it is essential to consider these potential limitations when attempting to develop biocatalytic processes. To address this issue, enzymes can be engineered to be more selective by altering the active site of the enzyme or by introducing allosteric regulation. Additionally, enzymes can be used in combination with substrate-binding proteins to increase their selectivity. Biocatalysis selectivity is enhanced by localizing the enzyme reaction to a specific compartment or environment (Zakharchenko et al. 2018). Gene expression regulates enzyme concentration chemically. For example, the composition and sugar content of the fruit varies according to species, stage of development, and variety. Genetic control of sugar metabolism to improve fruit quality is essential (Desnoues et al. 2016). Therefore, biocatalysis selectivity can be improved by regulating gene expression according to the environment, resulting in improved fruit quality.

### Potential probiotics and starters

A probiotic is a live microorganism that provides health benefits to its host when administered in sufficient amounts. In order to grow and form colonies in the digestive tract, probiotics must be resistant to stomach acid and bile (Saad et al. 2013). It is generally understood that probiotics are derived from a heterogeneous group of LAB (Lactobacillus, Enterococcus) and Bifidobacterium genera, such as lactic acid bacteria and those commercially available for human consumption. Although yeasts play a major role in food and are widely distributed, they have not yet been well investigated as potential probiotic candidates. As probiotics, yeasts and other microbes are also being developed (Kim et al. 2019; Suez et al. 2019). Foods containing such bacteria belong to the functional foods category, which describes foods that have a health benefit. In the 1980s, Japan first used the term "functional food" to describe foods enriched with ingredients that have physiologically beneficial effects (Topolska et al. 2021). As well as producing proteinaceous antimicrobial agents, LABs also produce bacteriocins. Bacteriocins are peptides that exhibit antibacterial activity against food spoilage organisms and foodborne pathogens but do not affect producer organisms (Moradi et al. 2020). Clinical relevance of probiotics was first reported in the literature for the treatment of diarrhea, ulcerative colitis, and pouchitis. There are two critical factors when choosing a probiotic candidate regarding potential health benefits. Viability and quantity upon ingestion, survival and stability in the digestive system (Di Cagno et al. 2020). Vera-Pingitore et al. (2016) identified strain *L. plantarum* Q823 as a viable probiotic candidate for use during fermentation of quinoa-based beverages. Despite growing in quinoa-based products, this strain survives and colonizes the human GI tract. *Akkermansia muciniphila* has attracted excessive attention recently and is considered a potential probiotic because other studies demonstrated a causal relationship with obesity, inflammation, cancer, and metabolic abnormalities (Dao et al. 2016). As integral commercial starter cultures, LABs play an essential part in food fermentation by breaking down carbohydrates into more minor metabolites, including lactic acid, acetic acid, or carbon dioxide. Fermented food manufacturers can get starter cultures readily available in a highly concentrated form or create a custom culture. Selecting the proper factory is determined by the quantity of products that need to be produced. Factors such as the level of automation, microbiological expertise, production costs, and economic considerations all come into play.

Additionally, microbial starter cultures play a crucial role in maintaining a product's quality and functionality, including taste, texture, pH, and alcohol content (Bachmann et al. 2015). Industrial food fermentation uses lactic acid yeast, and ongoing research is focused on improving them (Table 2). A starter medium should have desirable properties such as durability in process, rapid growth, high biomass and yield, and specific organoleptic characteristics (Smid and Kleerebezem 2014).

**Table 2:** Fermentation starter microorganisms in various products

Fermented products’ name	Bacteria involved in fermentation	Type of fermentation	Place/origin consumption	References
Kefir	Lactic acid bacteria (LAB)	Starter culture	Caucasus	Lopitz-Otsoa et al. (2006)
Sucuk	Lactic acid bacteria (LAB)	Starter culture	Türkiye	Bingol et al. (2014)
Kimchi	Lactic acid bacteria (LAB), Leuconostoc, Weissella, and Lactobacillus	Spontaneous, added commercially	Korea	Noh et al. (2016)
Sourdough bread	Yeasts, lactic acid bacteria (LAB), and acetic acid bacteria (AAB)	Spontaneous, starter, added commercially	Middle East and Europe	Landis et al. (2021)
Nham	*P. pentosaceus* and *L. Namurensis*, Lactic acid bacteria (LAB) and nitrate-reducing bacteria	Starter culture	Thailand	Kantachote et al. (2015)
Tempeh	*R. oligosporus*	Starter culture, backslopping	Indonesia	Ahnan‐Winarno et al. (2021)

### Utilization of extremophiles in fermentation

The term “extremophile” describes organisms that survive under challenging environmental circumstances (temperature, pH, salinity, and pressure) and is receiving significant interest due to their capacity for catalyzing reactions and the possibility of practical utilization in extreme environments (Gupta et al. 2014). Among the substrates they address are acidophilic, alkaliphilic, halophilic, xerophilic, thermophilic, psychrophilic, methylotrophs, and gaseous substrates (Adebo et al. 2017). It is imperative that a practical approach to controlling aflatoxin B1 (AFB1) is developed, as its presence in food can be a serious health concern. Thermophiles can also contaminate food. Powdered milk products can be contaminated by *Anoxybacillus flavithermus* and *Geobacillus* spp. (Irwin 2020). Lactase activity is lacking in *Geobacillus thermoglucosidasius* and it grows slowly in fat-free milk, dependent on *A. flavithermus* presence to provide them with glucose and galactose for growth (Zhao et al. 2018). They deliver appropriate nutrients and other substances, such as beta-carotene, which can be used as a pigment or to provide vitamin A, as do Halophiles.

Furthermore, they can spoil highly acidic foods with low water activity, such as *Debaryomyces hansenii*, an extremophilic yeast. As *D. hansenii* outcompetes unwanted microorganisms for nutrients and produces antimicrobial metabolites, both extracellularly and intracellularly, it inhibits the germination of *Clostridium butyricum* and *C. tyrobutyricum* in cheese brines. The ability of *D. hansenii* to multiply in cheese and its ability to consume lactate, citrate, lactose, and galactose make it an excellent starter culture for producing cheese.

Furthermore, several reports indicate that D hansenii enzymes may be active in meat fermentation, but they had minimal influence on the formation of volatile substances essential for aroma production in garlic-flavored fermented sausages and model mince (Krüger et al. 2018). In fermented foods, extremophilic lipases and esterases hydrolyze glycerols and fatty acids to produce polyunsaturated fatty acids. Additionally, piezophilic extremozymes are also useful for fermented food products that require high pressure (Zhang et al. 2015).

## Fermented dairy products

Fermented foods, especially dairy, represent a rapidly expanding sector of the global food market, making it imperative to recognize and understand dairy fermented foods in the past and present (Kaur et al. 2022). The word “ferment” derives from Latin “fervere”, which means to boil, and is defined as “the chemical breakdown of a substance by bacteria, yeast or other microorganisms, typically by foaming and releasing heat”. Fermented foods are the products of controlled microbiological growth and enzyme transformations of food ingredients, and fermentation is a deliberate and controlled method of achieving desired qualities (Marco et al. 2021). Fermentation is the breakdown of organic molecules into simpler forms by microorganisms (Sharma et al. 2020). By introducing certain microorganisms, fermentation can produce desired flavors and aromas, as well as enhance the nutritional value and shelf-life of foods. In addition, fermentation can help preserve foods by producing antimicrobial compounds, such as lactic acid, which can prevent spoilage.

Fermented dairy products fall into two categories: traditional and non-traditional (Kroger et al. 1992). Conventional fermented dairy products were first produced approximately 10,000–15,000 years ago, when human lifestyle changed from gathering food to producing it (Bintsis and Papademas 2022). This change was due to the domestication of animals and the use of their milk as a food source. The fermentation process was used to preserve the milk and make it more palatable. This allowed for the production of a variety of dairy products such as cheese, yogurt, and kefir. Societies during the Neolithic period consciously preferred to consume different animals' milk for cultural and taste reasons and processed this milk in various ways (Charlton et al. 2019; Salque et al. 2013). Traditional fermentation has been employed for centuries in raw milk processing. The process is spontaneous, and part of the fermented product is used to inoculate the new batch (Galli et al. 2022). In contrast, non-traditional fermented milk products have recently been developed. These products are produced with known microbial cultures based on scientific principles, and their quality can be optimized (Galli et al. 2022; Kroger et al. 1992). Non-traditional fermented milk products are more consistent in quality as the addition of known microbial cultures creates a more controlled fermentation process. This process also ensures that the products are standardized and free of any potential health risks associated with raw milk. It has been reported that probiotic-based fermented functional foods are becoming increasingly popular since the early 2000s (Kaur et al. 2022). From the past to the present, fermentation practices have been influenced by various factors such as raw materials, climatic conditions, production area, social, cultural, religious, and economic aspects (Galimberti et al. 2021). These factors have helped to shape the diversity of fermented products, and have also helped to influence the consumption of probiotic-based fermented functional foods. The popularity of these foods has been further strengthened by the health benefits associated with them, such as improved digestion, increased nutrient absorption, and enhanced immunity. Milk and dairy products are now consumed worldwide, primarily in pasteurized and fermented forms. However, variations in consumption rates are caused by per capita income and the impact of regional preferences (Muunda et al. 2023). This is due to the fact that those with higher incomes can afford to purchase more nutrient-rich foods and have access to a variety of different ingredients to choose from. Additionally, regional preferences play a significant role in the demand for certain food items, as people’s taste and cultural preferences vary from one region to another.

Fermented foods with live microorganisms include yogurt, kefir, cheeses, miso, natto, tempeh, kimchi, kombucha, and some beers (Voidarou et al. 2021). Some foods are subjected to pasteurization, smoking, baking, or filtering after fermentation, causing live microorganisms to die or be removed. Sourdough bread (baked), shelf-stable pickles (heated), sausages (heated), soy sauce (heated), vinegar (heated), most beers, distilled spirits (filtered), coffee and chocolate beans (roasted) are fermented products (Li et al. 2022). Still, microorganisms have died or been eliminated from fermentation. Foods such as fresh sausages, vegetables preserved in brine or vinegar, processed soy sauce, non-fermented dried meats and fish, and acidified cottage cheese are not considered fermented, as live microorganisms are not involved in production. Fermented foods are sometimes called “probiotic foods” or “probiotics” and are used interchangeably. However, using these definitions interchangeably is incorrect (Marco et al. 2021). Probiotics contribute to their beneficial effects when administered in sufficient quantities. They do not have to take a specific form to have a positive effect on the host. Probiotics are live microorganisms that have a beneficial effect on the host, while fermented foods are simply foods that have gone through a process of fermentation (Dahiya and Nigam 2022). The probiotic benefits of fermented foods come from the live microorganisms present in the food, which are not always present in sufficient quantities to have a positive effect on the host. Molecular components of probiotic-containing foods show prophylactic or therapeutic effects against disease-causing agents. These foods are generally known as nutraceuticals, foodiceuticals, functional foods, or medifoods (Kaur et al. 2022). These foods interest consumers based on their nutritional and organoleptic properties and beneficial effects on human health (Luz et al. 2021). The effects of these foods are attributed to the presence of bioactive compounds, which can be of plant or microbial origin. These compounds, such as antioxidants, polyphenols, vitamins, and minerals, have protective effects against disease-causing agents like bacteria and viruses.

The fermentation process produces large quantities of lactic acid, alcohol, or acetic acid that inhibit other microorganisms. They also continue to reproduce unaffected by these generated substances, a process known as “amensalism” (Teng et al. 2021). These by-products generated by the fermentation process are toxic to other microorganisms, making them unable to reproduce. This gives the fermenting microorganisms a competitive advantage, allowing them to outcompete other microorganisms in the environment. Fermented products are usually thicker than milk because acid precipitates milk proteins. Pathogens are inhibited by high acidity and low pH (Kumar 2017). Fermented dairy products have a unique, desirable flavor, texture, aroma, and improved digestibility compared to the raw materials they produce (Bintsis and Papademas 2022). However, the wrong fermentation process poses a health hazard. Unhygienic conditions or improper food production lead to contamination and spoilage. Foodborne disorders are brought on by spontaneous fermentation by unidentified microbes, which promotes the growth of undesired and even hazardous microorganisms (Teng et al. 2021). This can cause food to be unsafe to eat, leading to food poisoning. Symptoms of food poisoning can range from mild to severe, and can even be life-threatening. Therefore, it is important for food producers to take proper steps to prevent contamination and spoilage.

Propionic acid bacteria (PAB) and lactic acid bacteria (LAB) are microorganisms utilized to make cheese and other fermented dairy products. LAB is used to acidify milk, and PAB is used for its aromatizing properties (Thierry et al. 2015). Propionic acid bacteria are microorganisms that produce propionic acid and are involved in producing fermented propionic cheeses, such as Swiss cheese, with exceptional adaptability to technological and physiological stress conditions. The propionic acid fermentation in cheese causes characteristic pores, cracks, and a slightly sweet flavor (Antone et al. 2022; Bücher et al. 2021). Propionic acid bacteria are also responsible for the formation of carbon dioxide during the fermentation process, which gives cheese its airy, spongy texture. This also contributes to the flavor of the cheese, as the carbon dioxide imparts a slightly sour taste. Propionic acid bacteria metabolism differs significantly from lactic acid microorganisms. It is characterized by the production of carbohydrates during fermentation, except for lactic acid, propionic acid, and acetic acid (Vakhrusheva et al. 2022). As a result of PAB's metabolic activities, the product is enriched with organic acids, vitamins (B2, B12, K, and folate), and other nutrients, increasing the stability and nutritional value of food products (Antone et al. 2022). Fermented dairy products provide an ideal environment for probiotic bacteria to grow in the human gut. LAB include *Lactobacillus*, *Streptococcus*, *Lactococcus*, *Bifidobacterium*, *Leuconostoc*, *Enterococcus*, and *Pediococcus*, which are among the most common strains of probiotic bacteria found in fermented dairy products (García-Cano et al. 2019; Kaur et al. 2022). In addition, yeasts and molds such as *Debaryomyces, Kluyveromyces, Saccharomyces*, *Geotrichum, Mucor, Penicillium*, and *Rhizopus* species are employed as fermenting microorganisms (Sharma et al. 2020). Fermented milk products are prepared using different starter cultures, and the types of microorganisms used in production are specified in the regulations (Table 3). Fermentation preserves probiotic properties while maintaining microbial viability and production (García-Cano et al. 2019; Kaur et al. 2022)*.* This helps to ensure that the fermented milk products are safe to consume and that they have the desired probiotic properties. This is because the starter cultures help to control the growth of unwanted microorganisms while promoting the growth of beneficial ones.

**Table 3:** Microorganisms used as starter cultures in fermented milk products (CAC 2003)

Fermented dairy products	Microorganisms used fermented milk products
Yoghurt	*Streptococcus thermophilus* *Lactobacillus delbrueckii* subsp.* bulgaricus*
Alternate culture yoghurt	*Streptococcus thermophilus* *Lactobacillus* spp.
Acidophilus milk	*Lactobacillus acidophilus*
Kefir	Starter culture prepared from kefir grains *Lactobacillus kefiri* *Lactobacillus kefiranofaciens* *Lactobacillus kefirgranum* *Kluyveromyces marxianus* *Saccharomyces* spp. *Lactobacillus* spp. *Leuconostoc* spp. *Lactococcus* spp. *Acetobacter* spp.
Kumys	*Lactobacillus delbrueckii* subsp. *bulgaricus* *Kluyveromyces marxianus*

Due to their resistance to low pH, adaptability to milk and other foods, and GRAS (generally recognized as safe) status, *Lactobacillus* species are also widely used (Galli et al. 2022). LAB are intentionally added to the product as starter cultures to reduce the ripening period and improve sensory characteristics such as color, flavor, aroma, and texture (García-Cano et al. 2019). Furthermore, food digestibility and product safety are enhanced by LAB fermentation due to the inhibition of spoilage and pathogenic bacteria. Certain LAB strains are considered probiotics because of their positive effects on the gastrointestinal system and human health and their significant roles (García-Cano et al. 2019). Lactic acid bacteria regulate the release of fatty acids. These properties of LAB involve metabolic processes involving enzymes such as lipases, proteases, and antibacterial proteins (Galli et al. 2022). Fermentation of yogurt and cheese results in a pH decrease due to the synthesis of metabolites such as organic acids, lactic acid, hydrogen peroxide, bacteriocin, diacetyl, acetaldehyde, and reuterin (Akbar et al. 2019).

Lactic fermentation and alcoholic fermentation are the two main types of fermentation carried out by microorganisms in fermented milk products, respectively (Galli et al. 2022). In lactic fermentation, lactic acid bacteria are the dominant species. Lactic fermentation products are classified according to LAB characteristics as mesophilic fermented milk, such as buttermilk, thermophilic products, such as yogurt, acidophilic milk, and probiotic products. Alcoholic fermentation occurs in products such as kefir, kumys, and Viili by yeasts and lactic acid bacteria (Galli et al. 2022). Microbial cultures, particularly those having proteolytic activity, are frequently employed in the dairy industry to produce cheese, yogurt, kefir, and other so-called fermented milk products. In manufacturing cheese, proteolytic enzymes are used to coagulate milk proteins and hydrolyze proteins. Proteolytic enzymes extract protein hydrolysates from milk to produce easily digestible dairy products (Kieliszek et al. 2021). Proteases produced by lactic acid bacteria used in lactic fermentation are capable of reducing milk protein allergens, depending on the strain and the proteolysis process. Lactic acid bacteria are a good source of hydrolyzing allergenic proteins in milk, and one isolate (*Enterococcus faecalis* VB43) was reported to be an excellent potential agent for the production of hypoallergenic dairy products (Biscola et al. 2018). Lactic acid bacteria use β-galactosidase to hydrolyze lactose into glucose and galactose. The hydrolysis of lactose lowers the intestinal pH and promotes the production of lactic acid, which inhibits the growth of microorganisms that cause putrification (Gholamhosseinpour and Hashemi 2019; Sharma et al. 2020). Furthermore, lactic acid is essential for calcium absorption and developing organoleptic properties (Sharma et al. 2020). The bioavailability of minerals such as calcium, potassium, zinc, magnesium, magnesium, potassium iodide, and phosphorus is increased due to the fermentation process of lactic acid bacteria and acidity (García-Burgos et al. 2020).

Several studies are currently being carried out that may be useful for isolating new probiotics and developing fermented milk products with probiotic properties. Luz et al. (2021) examined seven LAB strains isolated from breast milk for their probiotic properties and used *Lactobacillus plantarum* 5H1 and *Lactobacillus plantarum* 5L1 strains in the production of probiotic fermented milk. LAB strains have a wide range of antimicrobial activity against pathogenic bacteria and toxicogenic fungi. During the milk fermentation process, an increase in lactic acid content, a decrease in milk pH, and an increase in total bacterial count were observed. During storage, LAB viability in fermented milk remained at 8-log_10_ CFU/mL. *Lactobacillus plantarum* 5H1 and *Lactobacillus plantarum* 5L1 exhibited significant antimicrobial activity, sensitivity to antimicrobials, a broad spectrum of enzymatic activity, adhesion to Caco-2 cells, and reduction of *Salmonella enterica* adhesion. Furthermore, these selected strains remained viable during fermented milk storage and fermentation at 4 °C. This indicates that these strains are extremely hardy and have the potential to be used as probiotics that can survive through the fermentation and storage process.

The ability of microorganisms in fermented milk products to benefit the host is dependent on the presence of sufficient numbers of probiotic microorganisms in various products as well as the ability of adequate numbers of live microorganisms to reach the human intestine (Farahmand et al. 2021; Ranadheera et al. 2012). Thus, the legislation specifies the minimum number of live microorganisms that must be present in fermented milk products. The total number of microorganisms forming the starter culture used in products named fermented milk, yogurt, alternate culture yogurt, acidophilus milk, kefir, and kumys should be at least 107 CFU/g, the number of yeasts should be at least 104 CFU/g, and the label should be at least 106 CFU/g (Mukherjee et al. 2022). The number of live probiotics during the shelf life of fermented dairy products varies depending on many factors. These factors include the temperature of storage conditions, hydrogen peroxide (H_2_O_2_) produced by other bacteria present, dissolved oxygen content due to processing conditions, pH of the end product, acidity, and strain variation. In particular, the decrease in pH during storage, the presence of dissolved oxygen, and the presence of preservatives in the final products are the major factors contributing to the loss of cell viability (Farahmand et al. 2021; Terpou et al. 2017). At the end of their shelf life, 22 of 36 commercial probiotic fermented milk products sourced from the UK and European markets (61.1%) contained more than 106 CFU/g of *Lactobacillus* strains in accordance with the minimum recommended therapeutic level for probiotics. Rep-PCR was used to differentiate the isolated strains using the GTG-5 primer, and the isolated *Lactobacillus* species were identified as *Lactobacillus acidophilus, Lactobacillus casei*, and *Lactobacillus paracasei* (Farahmand et al. 2021). Another study found many areas for improvement in the number of cultures and accuracy of label information in commercial kefir products. More qualified controls of fermented foods are needed to demonstrate and understand their potential health benefits for humans. Consumers should demand higher levels of accuracy and quality, and regulatory bodies should conduct regular checks on these products (Metras et al. 2021). The antibacterial activity of LAB in fermented milk samples against *Salmonella typhimurium, Staphylococcus aureus, Escherichia coli*, and *Listeria monocytogenes* was determined. *L. lactis* subsp. *lactis* had a broader antimicrobial spectrum than the other isolates, and the probiotic evaluation of *L. lactis* showed that it could survive at low pH (pH 3) and 0.3–3% bile salts. It was concluded that LAB with antimicrobial activity is promising against food spoilage and pathogenic microorganisms in foods (Akbar et al. 2019). Hikmetoglu et al. (2020) reported that the microbial content (*Lactobacillus* spp., *Lactococcus* spp., *Lactobacillus* *acidophilus*, *Bifidobacterium* spp., and yeasts) of traditional kefir increased during fermentation and did not change significantly during cold storage of 7 days. Lactose content decreased during fermentation, while lactic acid gradually increased and remained constant during storage. Galactooligosaccharides in kefir samples were found to be stable during storage. The major LAB species isolated and identified from traditional fermented milk in Ghana based on 16S rRNA gene sequencing were *Ent. faecium, Lb. fermentum, Lb. plantarum*, and *Pd. acidilactici* (Motey et al. 2021).

Consumers can choose from a variety of fermented milk products. There are a few homemade products among them, but most are manufactured industrially. A variety of fermented milks and derivatives have been developed around the world, each with its own history. The type of milk used, the pre-treatment of the milk, temperature, fermentation conditions, and subsequent technological processes greatly influence their nature. The type of milk used can affect the flavor, texture, and overall characteristics of the fermented milk product. Pre-treatment of the milk can also influence the flavor, such as pasteurization or homogenization. Temperature and fermentation conditions can also have an effect, such as the length of fermentation and the type of starter culture used. Finally, the post-treatment processes, such as packaging and storage, can influence the shelf life and other characteristics of the product. Curd, yogurt, cheese, and kefir are among the most popular dairy foods (Kumar 2017).

Curd is a dairy product obtained by souring milk or decomposing it after adding any acidic substance. In some cases, it can also be made by mixing milk with acidic substances like lemon juice or vinegar. The liquid part is whey, and the solid part is curd. Whey contains the whey proteins of milk, while curd comprises milk proteins or casein (Kumar 2017). Traditionally, the curd is prepared from raw milk or boiled milk. Raw milk can undergo natural fermentation without adding any microorganisms, while boiled curd is prepared by inoculating boiled milk from the previous batch for fermentation (Joishy et al. 2019). It was determined that the main bacterial genera in the curd obtained from boiled milk were *Lactobacillus, Leuconostoc, Lactococcus*, and *Acetobacter*. In contrast, the leading bacterial genera in the curd obtained from raw milk were *Chryseobacterium, Enterococcus, Lactobacillus, Leuconostoc, Lactococcus, Streptococcus, Klebsiella, Acinetobacter, Pseudomonas,* and *Enhidrobacter.* *Lactococcuslactis* subsp. *cremoris* dominated the curd obtained from both raw and boiled milk. Moreover, several metabolites such as 10-methyl dodecanoic-5-olide, ascorbic acid, and 2,2,4-trimethyl-1,3-pentanediol diisobutyrate were significantly higher in curd produced from raw milk, while dodecanoic acid and glycerol 2-acetate were substantially higher in curd produced from boiled milk. *Lactobacillus* strain and metabolites detected in curd samples were farm-specific (Joishy et al. 2019).

Yogurt is milk's lactic fermentation product. Lactic acid fermentation is the process by which lactic acid bacteria convert milk sugar, lactose, into lactic acid, lowering the pH. Acidification is a key mechanism responsible for coagulation during yogurt fermentation (Muncan et al. 2020). The casein proteins that make up the gel matrix are responsible for the thick structure of the yogurt after it has been coagulated. Casein micelles in milk exist as a colloidal calcium caseinate phosphate complex. The acidification of the milk, i.e., the lowering of the pH, leads to the dissolution of the colloidal calcium phosphate and the release of the casein content from the milk. First, at the beginning of acidification, when the pH value is reduced from 6.7 to 6, only a small amount of colloidal calcium phosphate is dissolved, so structural changes in micelles are limited. When the pH is reduced to 5, the colloidal calcium phosphate is completely dissolved, and when the pH drops to the isoelectric point of casein (pH 4.6), casein micelles aggregate and form the yogurt gel matrix (Muncan et al. 2020). The yogurt cultures *Lactobacillus delbrueckii* subsp. *bulgaricus* and *S. thermophilus* have a symbiotic relationship and produce yogurt with an excellent taste, acidity, and viscosity. *L. delbrueckii* subsp. *bulgaricus* can readily utilize pyruvic acid, formic acid, folic acid, and long-chain fatty acids produced by *S. thermophilus*, while *L. delbrueckii* subsp. *bulgaricus* contributes to *S. thermophilus* growth (Dan et al. 2017). Furthermore, the volatile compounds produced by lactic acid bacteria significantly influence the flavor of the products. The flavor comprises a variety of volatile compounds, including acids, aldehydes, alcohols, ketones, esters, and aromatic hydrocarbons, and each class has its organoleptic properties (Dan et al. 2017; Farag et al. 2022). Yogurt is not a probiotic food, but it contains non-probiotic bacteria from milk fermentation. However, probiotic yogurt can be obtained by adding probiotic bacteria. Also, yogurt products obtained from a mixture of probiotics (*L. rhamnosus* GG) and prebiotics (dietary fiber) can be received (Kaur et al. 2022). The probiotic bacteria in yogurt contain beneficial bacteria that can help to support the good bacteria already present in the body. They can help to restore the balance of bacteria in the gut, which can help improve digestion and overall health. Prebiotics, on the other hand, are dietary fibers that act as food for the beneficial bacteria, helping them to thrive and grow in the gut. Therefore, combining probiotic bacteria with prebiotics can help to create a more beneficial environment in the gut.

Kefir is acidic-alcoholic fermented milk formed by the fermentation of milk by cauliflower-like kefir grains or starter microorganisms containing lactic acid bacteria, acetic acid bacteria, and yeasts, and is different from other fermented products due to the specificity of starter microorganisms (Al-Mohammadi et al. 2021; Bengoa et al. 2019; Fatahi et al. 2021). Kefir grains are white or light yellow in color, elastic in consistency, 0.3 to 3.5 cm in diameter, and composed of protein and polysaccharides (Al-Mohammadi et al. 2021; Bengoa et al. 2019). Kefir grains comprise approximately 83% water, 4 ± 5% protein, and 9 ± 10% a polysaccharide called kefiran (Bengoa et al. 2019). During fermentation, lactic acid, acetic acid, ethanol, carbon dioxide, organic acids, amino acids, vitamins (E, B3, B6, and B12), minerals (Se, Fe, Zu, and Mn), and enzymes (glutathione peroxidase, catalase, and superoxide dismutase) are formed (Fatahi et al. 2021). The pH of kefir ranges between 4.2 and 4.6, the ratio of ethanol between 0.5 and 2.0%, the ratio of lactic acid between 0.8 and 1%, and the ratio of CO_2_ between 0.02 and 0.2% (Rosa et al. 2017). Kefir inhibits pathogenic bacteria and fungi, and the inhibitory activity is more robust against bacteria (Al-Mohammadi et al. 2021). The antimicrobial activity of kefir and probiotic yogurt samples produced from cow, camel, sheep, and goat milk was investigated. It was determined that kefir samples had more substantial antifungal and antibacterial effects than probiotic yogurt samples, and kefir and yogurt samples produced from sheep and cow milk showed the highest and lowest antimicrobial activity against *S. aureus, E. coli, S. enterica,* and *L. monocytogenes*, respectively. *A. niger, S. aureus,* and *L. monocytogenes* were the most susceptible microorganisms, while *Penicillium* spp*. *and *E. coli* were the most resistant. Bioactive substances, organic acids, ethyl alcohol, hydrogen peroxide, diacetyl, peptides, possibly bacteriocins, and other inhibitory compounds have been suggested to be responsible for inhibiting the growth of pathogenic bacteria (Azizkhani et al. 2021).

Kumys is a fermented milk product made by fermenting mare's milk in two stages with bacteria and yeasts: lactic fermentation and alcoholic fermentation. The mare’s milk to be used in kumys production should have an acidity of ≤ 0.06% lactic acid, a density of 1029–1033 g/mL, and a fat content of at least 1% (Kondybayev et al. 2021). Fermentation is 1–3 days at about 20 °C (Liu et al. 2019). The consistency of kumys is a liquid, homogeneous, carbonated, slightly foaming drink without any fat particles (Kondybayev et al. 2021). The products of kumys fermentation are lactic acid, ethyl alcohol (0.6–3%), carbon dioxide, and other by-products such as volatile acids, alcohols, and other compounds with strong and distinctive aroma and taste (Afzaal et al. 2021; Kondybayev et al. 2021). According to the lactic acid content, three types of kumys are defined: strong, medium, and light (Afzaal et al. 2021). The fermentation time of kumys does not affect its quality. Milk from fresh mares and mature kumys had the highest chemical and nutritional content, while immature kumys (fermentation time less than 9 h) was not good quality (Liu et al. 2019). The microbiota of raw mare’s milk has a higher microbial diversity than that of kumys. Raw mare's milk is rich in LAB, such as *Lactobacillus helveticus, Lactobacillus plantarum, Lactococcus lactis,* and *Lactococcus kefiranofaciens*. In contrast, raw mare's milk contains pathogenic bacteria such as *Staphylococcus succinus, Acinetobacter lwoffii, Klebsiella oxytoca,* and *Klebsiella pneumoniae.* The change in microbiota composition and structure could be attributed to the transition from a slightly alkaline environment in raw mare's milk to a highly acidic kumys environment. The acidic environment in kumys inhibited the growth of most environmental pathogens, increased food hygiene, and minimized the risk of infection by endogenous pathogens found in raw mare’s milk (Zhang et al. 2020b).

Fermented foods are also valued for their improved shelf life, safety, nutritional value, and other properties, and are the most widely consumed by humans (Walhe et al. 2021). Fermented dairy products have several health benefits when consumed regularly and in a balanced and appropriate proportion (Kaur et al. 2022). The various health benefits of yogurt, cheese, kefir, and other traditional fermented milk products have been extensively researched. Probiotic strains of bacteria present in fermented dairy products are beneficial for gut health and can reduce the risk of certain diseases (Okoniewski et al. 2023). Additionally, fermented dairy products are a rich source of vitamins and minerals, including calcium, potassium, phosphorus, and B vitamins. Fermented dairy products are an effective treatment method that contains natural ingredients with high nutritional and digestibility, anti-hypertensive, hypo-cholesterolemic, antioxidant, immunomodulatory, and anti-inflammatory properties and fewer adverse side effects (García-Burgos et al. 2020). Probiotic consumption has been shown to positively affect the reduction of ailments from diarrhea to cancer (Akbar et al. 2019). Regular kefir consumption has been linked to benefits for lactose intolerance and the digestive system, as well as antibacterial, antihypertensive, anti-inflammatory, hypo-cholesterolemic effects, plasma glucose control, antioxidant, anticarcinogenic, antiallergenic activity, and wound healing (Rosa et al. 2017). Koskinen et al. (2018) found that low-fat fermented dairy consumption was inversely correlated with cardiovascular disease risk. However, cardiovascular disease risk has been reported with very high consumption of unfermented dairy products and milk. Zhang et al. (2020a) stated that fermented dairy products reduce cardiovascular disease risk. (Lee et al. 2020) reported that *Lactobacillus plantarum* B719 can be used as an alternative in treating primary postmenopausal osteoporosis. (Fatahi et al. 2021) At 24 and 48 h, the interactions between different concentrations of kefir drink and U87 cancer cells (glioblastoma), the most severe form of brain tumor, were evaluated. As a result, it was discovered that kefir significantly reduced the growth rate of U87 cells at increasing concentrations and had a killing effect. It could be used as a complementary treatment.

Companys et al. (2021) reported that consuming fermented dairy products reduces the risk of stroke and cardiovascular diseases, and consuming yogurt reduces the risk of developing type 2 diabetes mellitus. However, there is insufficient evidence that fermented milk or cheese consumption protects against metabolic syndrome. It is stated that the available evidence on the effect of dietary cheese and yogurt on hypertension is limited and that consumption of smoked cheeses should be limited in hypertensive patients due to their high sodium content. Walhe et al. (2021) isolated three isolates with probiotic potential (*Enterococcus faecium* (EF), *Enterococcus faecium* (Chole1), and *Lactobacillus pentosus* (7MP) from yogurt and determined that among these three isolates, *Enterococcus faecium* (EF) and *Enterococcus faecium* (Chole1) produced vitamin B12 in a fair amount (1 ng/mL); whereas, *Lactobacillus pentosus* (7MP) had the highest cholesterol reduction potential (48%) compared to the others.

## Fermented meat products

Meat fermentation is an ancient preservation method widely used to increase meat products’ taste, aroma, palatability, color, tenderness, and shelf life. Meat is exposed to microorganisms or enzyme activities during fermentation, so desirable changes occur in meat biochemistry. The process of fermentation causes proteins and fats to break down, resulting in a more tender meat product and a more intense flavor (Wang et al. 2022). The breakdown of proteins and fats also helps to protect the meat from spoilage, thus increasing its shelf life. Meat physicochemical, biochemical, and microbiological changes run the fermentation process and support the formation of desirable meat products (Kumar et al. 2017). The fermentation process is an essential metabolic process converting carbohydrates into acids, gases, and alcohol, resulting in the conversion of raw meat to fermented meat products through the activities of “cultured” or “native” microorganisms (Kumar et al. 2017). This is because the anaerobic environment formed during the fermentation procedure encourages the growth of some lactic acid bacteria strains (Ravyts et al. 2012). These bacteria strains produce lactic acid that gives fermentation products their distinctive flavor and texture. Lactic acid also acts as a natural preservative, extending the product's shelf life. Fermentation of meat products is performed by lactic acid bacteria on a “native culture” or “starter culture”. Using native flora for meat fermentation may cause many problems, including inconsistent quality. This problem was solved by cultivating a commercial starter culture in a controlled environment to maintain the same quality (Kumar et al. 2017). *Pediococcus cerevisiae* was the first starter culture for meat fermentation (Deibel et al. 1961). Then, the other species are *Lactobacillus sakei* and *Lactobacillus curvatus* among the LAB, *Staphylococcus xylosus*, and *S. carnosus* among the coagulase-negative staphylococci and *Debaryomyces hansenii* among yeasts were also used for meat product fermentation (Alessandria et al. 2014). LABs produce bacteriocins that improve meat quality and stability during fermentation. Bacteriocins are proteinaceous compounds that exhibit antibacterial activity against pathogens. Bacteriocins show bactericidal effects except for eukaryotic cells and are also tolerant of heat and salt (De Vuyst and Leroy 2007). This means that when these compounds are added to the fermentation process, they can help to inhibit the growth of harmful bacteria while also preserving the flavor of the meat. Furthermore, the heat and salt tolerance of bacteriocins ensures that the meat will remain safe for consumption for a longer period of time.

Sausages, dating back to 1500 BC, are the most popular and oldest meat products consumed globally because of their flavor and nutritional function. Generally, sausages are produced from salted minced or chopped meat. They are formed by filling seasoned raw meat with starter cultures into natural or artificial casings, then hanging them to ferment and ripen (Kumar et al. 2017). The starter culture is typically a single LAB species or a LAB mixed with other bacteria (*Staphylococcus xylosus* or *S. carnosus*) (Holck et al. 2017).

### Microbial spoilage in fermented meats

Microorganisms found in the meat microbiota interact with each other and meat substrate during processing and storage. A small percentage of these microorganisms growing on meat can deteriorate foods through their metabolic activities. For instance, lactic acid bacteria (LAB) produce lactic acid from meat carbon sources. Lactic acid produced by LAB has various positive and negative effects based on the kind of meat product (Lahiri et al. 2022). It also helps to form desirable changes in fermented meats, such as an acidic taste and decreased pH. However, it may cause undesirable changes in other products. Thus, microbial spoilage is a highly complex structure with varying properties depending on the microorganism, substrate, and the nature of the fermented product. Although several microbial metabolic pathways are known that lead to changes in the taste, color, odor, or texture of meat products and stimulate the generation of defined spoilage compounds, the primary mechanism that leads to spoilage still needs to be fully resolved. Therefore, to understand this mechanism that leads to meat spoilage, it is essential to initially comprehend the microbial factors of meat, their interactions, and metabolic activities (Zagorec and Champomier-Vergès 2023). To understand the primary mechanism that leads to meat spoilage, it is important to analyze the microbial components of meat, their interactions, and their metabolic activities. This will help to identify the pathways and mechanisms that lead to spoilage and can help improve food safety and quality.

### Mycotoxins in fermented meat

Meat and meat products can be contaminated with toxic compounds, such as mycotoxins, during production, storage, and distribution (Pleadin et al. 2021). Mycotoxins contamination in the final product can be related to raw materials, spices, additives used in production, or hazardous environmental components. Therefore, these toxic components that cause contamination can adversely affect human health (Pleadin et al. 2016). Mycotoxins are produced by certain types of fungi and can be found in crops, grains, and even in animal feed. When these contaminated raw materials are used in the production of meat and meat products, mycotoxins can be passed on to the final product.

Mycotoxins are secondary metabolites produced by molds, responsible for acute and chronic toxicity of humans and animals. Mycotoxin contamination can occur in meat products in these ways; due to meats supplied from animals fed with contaminated feed, the components such as contaminated spices added to the meat products, and can arise as a consequence of the activity of molds growing on the surface of fermented meat (Alapont et al. 2014; Bertuzzi et al. 2013). Ochratoxin A (OTA) and aflatoxin B1 (AFB1) are the most common mycotoxins contaminating fermented meat products. AFB1 is the most common liver carcinogen categorized in group 1 by the International Agency for Research of Cancer (IARC). *Aspergillus flavus* and *Aspergillus parasiticus* are known as responsible species for the production of AFB1. OTA is a group 2B carcinogen, and the genus of *Aspergillus* and *Penicillium* are responsible for its production (Pleadin et al. 2021). Mycotoxigenic molds isolated from fermented meat products can produce mycotoxins under various conditions, such as environmental temperature, humidity, and water activity during the ripening period of meat products (Pleadin et al. 2015). Several studies have pointed out the possibility of the presence of mycotoxin contamination in fermented meat products as a result of inadequate control of production and storage conditions, indicating the necessity of prevention of contamination that can adversely affect human health (Alapont et al. 2014; Pleadin et al. 2016). Pleadin et al. (2016) observed significant AFB1 and OTA levels in “Slavonski Kulen” fermented sausages as 11.79 ± 2.34 µg kg^–1^, 16.13 ± 3.32 µg kg^–1^, respectively. OTA levels in Istrian, Slavonian, and Kulenova Seka fermented sausages were determined as 0.25 ± 0.01 µg kg^–1^, 0.27 µg kg^–1^ and 0.26 ± 0.14 µg kg^–1^, respectively (Kudumija et al. 2020). In another study, OTA levels in İberian ham were noticed as 3.20 µg kg^–1^ (Rodríguez et al. 2015).

### Biogenic amines in fermented meat

Biogenic amines are organic compounds found naturally in many foodstuffs with an aliphatic, aromatic, or heterocyclic structure formed due to microbial decarboxylation of amino acids or the amination of aldehydes and ketones (Santos 1996). These compounds are formed by the action of microbial enzymes on amino acids, and they can contribute to the flavor, aroma, and texture of foods. They are also important as they can act as toxins, leading to food spoilage and safety issues.

It has been known that high-concentration exposure to biogenic amines can lead to toxic effects on respiratory and cardiovascular systems (Tsafack and Tsopmo 2022). Biogenic amines are more frequently found in fermented meat and meat products because of their predisposition to amine decarboxylation by the natural microbial flora. They may be produced during the fermentation stage by the activity of microorganisms while the meat proteolysis. Insufficient hygienic quality of raw material, re-contamination, and deficiencies in production and storage steps significantly impact the formation of biogenic amines (Gernah et al. 2011). In such circumstances, they are also used as a spoilage indicator and poor hygiene conditions for meat products. Lactic acid bacteria that have grown and displayed their metabolic activity on these fermented meat products have a crucial function in forming biogenic amines such as putrescine, cadaverine, histamine, and tyramine (EFSA 2011).

Biogenic amines have been reported numerous times in fermented meat products (Alves et al. 2017; Rabie et al. 2014; Sun et al. 2016). Alves et al. (2017) evaluated the biogenic amine levels of Portuguese and Serbian fermented dry sausages. While histamine was not detected in both sausages, cadaverine, putrescin, and tyramine were found significantly in these samples. Another study assessed biogenic amines in dry-ripened sausages made from different meats (horse, beef, and turkey). The study concluded that the total biogenic amine contents, from highest to lowest, were ranked for turkey, beef, and horse sausages, respectively. The high levels of total biogenic amines originated from the elevated histamine, putrescine, and tyramine content in turkey sausages (Rabie et al. 2014). Sun et al. (2016) observed the high histamine, tyramine, and cadaverine levels in traditional Chinese Sichuan-Style sausages (mean value of 196 mg/kg, 164.6 mg/kg, 141.6 mg/kg, respectively). Researchers indicated that these results were linked to poor hygiene of raw materials and insufficient hygiene conditions during processing steps. Due to the activity of microorganisms, fermented meat products contain higher levels of biogenic amines. Insufficient hygienic conditions were found to increase the amount of free amino acids present in the raw materials. These free amino acids are then available to be used by microorganisms during the fermentation process, leading to the increased biogenic amine contents of the final product.

### Fermented meat products worldwide

Raw meat is a highly perishable food, and preservation of meat is a significant problem. In early civilizations, preservation techniques such as salting and drying under the sun were used for long-term meat preservation. These preservation strategies resulted in lower water activity levels, which protected the meat from spoilage and pathogenic microorganisms (Zeuthen 2007). Many traditional fermented meat products are consumed by different cultures worldwide because of differences in raw materials, formulations, or manufacturing processes. The low water activity levels of the salted and dried meat provided a hostile environment for microorganisms, thereby preventing the growth of spoilage and pathogenic microorganisms. This allowed the meat to be stored for a longer period of time without it spoiling. Additionally, traditional fermented meats are processed differently in various cultures, resulting in different flavors, textures, and shelf lives. These products are an essential source of information about society's consumption habits. The quality and quantity of fermented meat products produced vary by country (Kumar et al. 2017). Table 4 lists some common fermented meat products.

**Table 4:** Some of the common fermented meat products worldwide

Origin/region	Product name	Substrate/raw materials	Fermented meat group	Microorganisms involved in fermentation	References
Germany	Teewurst	Pork, beef	Fermented sausage	LAB	Austin-Watson et al. (2013)
India	Kargyong Satchu	Pork or beef	Fermented sausage	LAB	Bhutia et al. (2021)
Italy	Piacentino, Crespone, Mortadella	Pork	Salami	LB	Baldin et al. (2018), Połka et al. (2015)
Italy	Prosciutto, Pancetta	Pork	Fermented meat	LAB	Parlindungan et al. (2021)
Portugal	Alheira	Pork or beef	Fermented sausage	LAB	Fernandes et al. (2022)
Spain	Androlla	Pork	Dry cured sausage	LAB and *Staphylococcus* spp.	Landeta et al. (2013)
	Chorizzo	Pork	Dry sausage	*Lactobacillus* and *Streptococcus* spp*.*	Juárez-Castelán et al. (2019), Prado et al. (2019)
Serbia	Sremska, Petrovská klobása	Pork and beef	fermented sausage	LAB	Milicevic et al. (2021), Vasilev et al. (2015)
Thailand	Nham, Goon chiang, Sai-krok e-san mu	Pork	Fermented sausage	LAB	Botthoulath et al. (2018), Wanangkarn et al. (2014)
Turkey	Sucuk, Pastırma	Beef	Fermented sausage dry-cured meat	LAB	Kamiloğlu (2022), Öz et al. (2017)

## Fermentation role in alternative proteins

### Fermentation of plant-based products

Fermentation of plant-derived products was used for many decades. From a historical perspective, during the Neolithic period in 8500–400 BC, fermentation accidentally started with plant-derived products, such as grain, to produce wine-like beverages, which can preserve plant-based products longer (Lavefve et al. 2019). Two thousand five hundred years ago, fermented beans were discovered to produce soy sauce Asian. The list of fermented plant-based products is found in multiple countries, as shown in Table 5. Last few decades, fermented plant-related food has become a novel food trend, although its popularity has decreased due to food industrialization, especially in European countries (Giacalone et al. 2022; Michel et al. 2021; Profeta et al. 2021). The interest in the last decades is due to the demand for healthy food products with healthy properties found in plant-based products (Anusha Siddiqui et al. 2022; Bryant and Sanctorum 2021; Schiano et al. 2020). For example, kombucha is produced from lactic acid bacteria, and acetic acid bacteria generate the product without alcohol, which is more acceptable for consumers in European countries.

**Table 5:** Fermented plant-based products

Fermented plant products	Plant-based source	Bacteria involved	Consumers attitude	Origin	References
Anishi	Taro (Yam) leaves	*B. subtilis* and *B. licheniformis*	Acted as a main ingredient in different food products	Nagaland, India	Jamir and Deb (2021), Mir et al. (2018)
Burong mustala	Mustard	*Pediococcus* sp*., L. brevis*	Consumed directly as pickles	Philippines	Di Cagno et al. (2013)
Cucumber	Cucumber	*L. pentosus, L. Plantarum, L. Brevis, Weissella* spp., *P. ethanolidurans, Leuconostoc* spp., and *Lactococcus* spp.	Consumed directly as pickles	United states, Asia	Moore et al. (2021), Pérez-Díaz et al. (2017)
Dakguadong / Pak-Gardong	Mustard leaves	*L. plantarum*	Consumed directly as pickles	Thailand	Di Cagno et al. (2013)
Dhamuoi	Cabbage	*Lc. Mesenteroides, L. plantarum*	consumed as a pickle	Vietnam	Di Cagno et al. (2013)
Ekung/Hirring	Bamboo tender shoots	*Lactobacillus plantarum, L. lactis, L. brevis, L. casei and T. halophilus*	Eaten raw and also by cooking with fish, meat and vegetables	Arunachal Pradesh	Sharma and Yaiphathoi (2020)
Fermented garlic	Garlic	*B. subtilis, B. methylotrophicus* and* B. amyloliquefaciens*	As condiment prepared with dressed olive oils	Spain	Chua et al. (2022)
Fermented peppers	Peppers	*Lactobacillus* spp., *Lc. citreum, Weissella cibaria, L. plantarum, L. paraplantarum, H. pseudoguilliermondii, K. ohmeri*	No effects of fermentation on the quality of the fermented peppers	Latin America and Caribbean	González-Quijano et al. (2014)
Goyang	Leaves of magane-saag (*Cardamine macrophylla* Willd.)	*L. plantarum, L. brevis, L. lactis, E. faecium, P. pentosus* and *candida* spp.	Consumed as a dish or with boiled noodles, meat and yak	Sikkim and Nepal	Mir et al. (2018)
Gundruk	Rayo-sag’, mustard leaves, and cabbages	*Pediococcus* and *Lactobacillus* spp*., L. cellobiosus* and *L. plantarum*	Consumed as a side dish and as an appetizer	Nepal	Tamang et al. (2005)
Inziangsang	Leaves of mustard	*L. plantarum, L. brevis* and *Pediococcus*	Consumed as a soup	Nagaland and Manipur	Tamang et al. (2005)
Khalpi	Cucumber	*L. plantarum, L. brevis* and *Leuconostoc fallax*	Eaten as pickle after mixing with mustard oil, salt and powdered chilies	Nepal	Tamang et al. (2005)
Kimchi / Sauerkraut	Cabbage, hot pepper, green onion, and ginger	*Lc. mesenteroides, L. brevis, P. cerevisiae, L. plantarum, Weissella* spp.	Consumed as a as pickle or soup	Korea, Europe, USA	Di Cagno et al. (2013), Kwak et al. (2014), Xiong et al. (2012)
Kombucha	Tea leaves	Lactic acid-producing bacteria, acetic acid-producing bacteria, and yeasts	Consumed as beverage and functional drink, related health	Eastern Asia	De Noronha et al. (2022), Lavefve et al. (2019)
Lung-Siej	Bamboo shoots	*Lc. fallax, L. mesenteroides, L. brevis, L. curvatus*, and *Lt. lactis*	Consumed as curry mixed with fish and meat	Meghalaya, North-eastern India	Nongdam (2015)
Mesu	Tender bamboo shoot	*L. plantarum, L. brevis, L. curvatus, Lc. citreum, P. pentosaceus*	Consumed as a pickle mixed with edible oil chillies	Darjeeling hills and Sikkim, North-eastern India	Tamang et al. (2005)
Olives	Olive	*L. plantarum*	Consumed as a pickle	USA, Spain, and middle-east countries	Hamid abadi Sherahi et al. (2018)
Oncom hitam	By products from tofu	*C. mesenterica, C. parapsilosis, C. parapsilosis* var*. intermedia, C. pelliculosa, C. reukaufii, C. solani, Mucor* sp*., M. javanicus, R. oligosporus,* and *R. oryzae,· C. mesenterica,* and* C. llarapsilosis*	As main dish consumed with rice	Indonesia	Ho (1986)
Salgam	Carrots	*L. plantarum, L. paracasei, L. fermentum, L. brevis*	Consumed as a pickle	Turkey	Di Cagno et al. (2013)
Sinki/Paocai	Radish-tap-root	*L. fermentum, L. brevis* and* L. plantarum*	Act as an effective appetizer	India, Nepal, and Bhutan	Yan et al. (2008)
Soibum	Bamboo shouts	*P. pentosaceus, L. brevis, L. plantarum, Lc. fallax, Lc. lactic*	Consumed as a side dish	Manipur, India	Mir et al. (2018)
Soidon	Bamboo shoots tips	*L. brevis, Lc. fallax* and* Lc. lactis*	Used as a curry by mixing with potato, green chillies, salt and fermented fish	Manipur, India	Jeyaram et al. (2010)
Soy sauce/miso/kecap	Soybean	*Bacillus, Aspergillus, Klebsiella, Cladosporium, Shimwellia, Aspergillus soyae*, *Zygosaccharomyces rouxii*, *Tetragenococcus halophilus*	Consumed as seasoning for food products	China, Indonesia and eastern Asia	Apriyantono et al. (1996), Cao et al. (2019), Fibri and Frøst (2019), Lee et al. (2017), Marco et al. (2017), Yang et al. (2017)
Tofu and tempeh	Soybean	*Bacillus* or LAB species, Lactic Acid Bacteria	Consumed with rice and other	Indonesia	Cao et al. (2019), Fibri and Frøst (2019), Ho et al. (2018)

Note: B.: *Bacillus, L.*: *Lactobacillus*, P.: *Pediococcus, Lc.: Leuconostoc*, *H.: Hanseniaspora, K.: Kodamaea, E.: Enterococcus, P.: Pediococcus, T.: Tetragenococcus.* LAB: Lactic acid bacteria, Lt.: *Lactococcus*, C.: *Candida*

Many fermented foods, as shown in Table 5**, **are a type of traditional foods which can raise the popularity of traditional food exposure to global consumers (Anusha Siddiqui et al. 2022). For example, pickling is fermented by immersing vegetables in vinegar into various foods to prolong their shelf life. The pickling system process looks like the fermentation of sauerkraut using lactic acid bacteria and salted brin performed at acidic fermentation (Di Cagno et al. 2013; Vitali et al. 2012). The world's most popular pickling is the small cucumber pickled vegetable, made with lactic acid bacteria, such as *lactobacillus* spp, *Weissella* spp*, Pediococcus* spp*, Pediococcus* sp*, Leuconostoc* spp*, and Lactococcus* spp*.* The fermentation in pickling can improve the taste of the pickling products. The pickling can be produced from other plant-based products, like olives (Hamid Abadi Sherahi et al. 2018) and carrots (Di Cagno et al. 2013). Other fermented plant-based products, such as fermented peppers and soybeans, have been reported. Peppers can be fermented with high levels of acetic acid, while soybean is fermented to produce various products, including soy sauce, tofu, and tempeh. Those fermented plant-based products involve different types of microorganisms.

Lactic acid bacteria, such as *lactobacillus* spp*.* play an essential role in the fermentation of food products (Di Cagno et al. 2013; Marco et al. 2017). Fermentation of vegetables typically happens accidentally, called spontaneous fermentation, where fermentation occurs due to lactic acid bacteria in the cabbage. Lactic acid bacteria initiated fermentation. Other species responsible for vegetable fermentation are *lactobacillus brevis, Leuconostoc mesenteroides, pediococcus,* etc. (Di Cagno et al. 2013; Xiong et al. 2012). For this species, culture-based methods are applied to initiate fermentation. The microorganisms involved during product fermentation determine the types of products. As an example, soibum and soidon are fermented products made from bamboo shoots in Manipur, India. Soibum involves the *P. pentosaceous* and *L. plantarum*, which are not used for fermenting Soidon products (Jeyaram et al. 2010; Yan et al. 2008). This also affects consumers' use of products. Soibum is typically served as a side dish, while Soidon is used as a curry mixed with potato and green chilies (Jeyaram et al. 2010; Mir et al. 2018). This difference in fermentation process and use of different ingredients results in a distinct flavor, texture, and aroma of Soibum and Soidon. As a result, consumers have different preferences for these two products, and they use them for different cooking preparations.

### Fermentation of fruits

Compared to plant-based food products, fermentation fruits are generally used as a beverage. Table 6 shows a list of fermented fruits with responsible bacteria. Fermented fruit products relate to a valuable and large beneficial microorganism. The majority of the time, different native microorganisms present in the raw components spontaneously ferment diverse plant-based substances to produce fermented products. The fermentation of fruits involves lactic acid bacteria, a small part of the microbiota of raw fruits (Di Cagno et al. 2013). Depending on the kind of vegetable, hetero- and homo-fermentative organisms from the genera *Pediococcus, Enterococcus, Weissella, Lactobacillus,* and *Leuconostoc,* were variably detected. The most prevalent species were *Weissella cibaria/Weissella confusa* and *Lactobacillus plantarum*. Fermented sweet cherries, for example, represent the alternative source of indigenous microorganisms (Di Cagno et al. 2011). The microbiota from the fruits is adapted to the fermented brine solution making the microorganisms isolated from an ecosystem typically having technological properties, such as resistance to salt, high acidification rates, pH, temperature, and phenolics (Di Cagno et al. 2013; Marco et al. 2017).

**Table 6:** Fermented fruits

Fermented fruits	Fruit source	Bacteria involved	Consumers attitude	Origin	References
Fermented cashew apple	Cashew apple	*L. mesenteroides*	Consumed as beverage	Brazil	Gupta and Abu-Ghannam (2011)
Pickled juice	Fruits	*L. buchneri*	Consumed as a pickle	China	Zeng et al. (2010)
Topache/Fermented pineapple pulp	Pineapple	*Meyerozyma caribbica*	Consumed as fresh	Mexico	Vitali et al. (2012)
Hardaliye	Grape	*L. paracasei, L. casei, L. pontis, L. brevis, L. acetotolerans, L. sanfrancisco,* and* L. vaccinostercus*	Stored in suitable containers at 4ºC and consumed either fresh or following aging	Turkey	Kabak and Dobson, (2011)
Fermented sweet cherry puree	Sweet cherry	*P. pentosaceus, L. plantarum*	Processed as brined, canned and frozen, dried or used for juices or syrups	Italy	Di Cagno et al. (2011)
Wine	Grape	*S. cerevisiae* and* S. bayanus*	Consumed as beverage	Georgia	Sahay (2019)
Cider	Apple	*L. brevis, L. paracollinoides, L. casei, L. diolivorans*	Consumed as beverage	UK	Di Cagno et al. (2013)

*L.*: *Lactobacillus*, *P.: Pediococcus, S.: Saccharomyces*

Other fermented fruits in Table 6 are also found in other countries with different fruit sources. Fermented cashew apples contain oligosaccharides, which can be evaluated with *Lactobacillus johnsonii* to determine the degree of polymerization. However, the fermented cashew apple is still considered to have an unpleasant taste to be consumed by humans (Vergara et al. 2010). The fermentation of fruit with *L. johnsonii* happens due to the focus on enzymatic synthesis, where glucose and fructose are used as enzyme acceptors. UK consumers drink cider, another beverage product from apples. This beverage is fermented by *L. brevis, L. paracollinoides, L. casei, L*. *diolivorans*. It is also under the same conditions as wine from grapes (Di Cagno et al. 2013). In general, fruit fermentation research is limited, whereas the fruits contain beneficial microorganisms to explore deeply to understand the importance of microorganisms isolated for the generated food products. Cider is produced by fermenting apples with certain species of lactic acid bacteria, which help to break down the fructose and glucose in the apples into ethanol. This fermentation process is similar to the process used to produce wine from grapes, although the microorganisms used are slightly different. As a result, more research is needed to better understand the role of microorganisms in fruit fermentation and their potential to generate beneficial food products.

### Fermentation of cereals

The majority of fermented foods manufactured from grains are found in Africa. The natural microbiota is employed to ferment grains like maize, millet, rice, or sorghum. The grains are frequently cooked, crushed, malted, and occasionally filtered. Many well-known cereal-based products have distinctive regional variations in content and preparation (Achi and Asamudo 2019; Tsafrakidou et al. 2020). African cereal products may be divided into a few main types; liquids, porridges, (semi)solid prepared doughs, and liquid drinks, such as nonalcoholic gruels (Achi and Asamudo 2019).

Table 7 lists fermented cereals and microorganisms responsible for fermentation. The Burkinabe dish of ben-saalga, a thin porridge made from fermented pearl millet (Pennisetum glaucum) sediment cooked in water, is well-known in Ghana (Achi and Asamudo 2019; Nout et al. 1995). Natural fermentation is often dominated by *L. fermentum, L. plantarum,* and *P. pentosaceus,* requiring the energy density of fermented gruels derived from cereal; some *L. plantarum* strains can hydrolyze starch, which can be advantageous. Another product is Ogi, a well-known morning gruel traditionally made by naturally fermented maize grains to create a supplementary diet for kids (Achi and Asamudo 2019; Gupta and Abu-Ghannam 2011; Nout et al. 1995). The precise composition will impact the end product's viscosity, fermentability, and content. It can also be prepared from sorghum or millet grains. Ogi is often ingested following heat treatment, eliminating the probiotic properties of lactic acid bacteria (Gupta and Abu-Ghannam 2011). It must be noted that these foods’ functional properties are connected not only to how bioactive live cells interact with the host but also indirectly by ingesting bioactive chemicals generated during fermentation. Thus, natural fermentation is a viable way to promote a healthy lifestyle through the consumption of plant-based foods with antimicrobial properties.

**Table 7:** Fermented cereals

Fermented cereals	Cereal source	Bacteria involved	Consumers attitude	Origin	Reference
Bensaalga, koko	Corn, pearl millet and red sorghum	*L. fermentum, L. plantarum,* and* Ped. pentosaceus*	Consumed as gruel	Burkina Faso, Ghana	Achi and Asamudo (2019)
Borde	Maize, barley, wheat, finger millet, sorghum	Lactic acid bacteria, such as *Lactobacillus* spp	Consumed as brown cultured beverage, with a dark consistency and a sweet–sour taste and consumed by adults and children often as a low-cost meal replacement	Ethiopia	Achi and Asamudo (2019), Hotessa and Robe (2020)
Boza	Wheat, millet, corn	Lactic acid bacteria, like *Lactobacillus* spp*.*, and yeasts including *Saccharomyces* spp*, Candida* spp	Consumed as beverage for consumers of all ages and is generally during winter months	Turkey and Balkan countries	Hancioǧlu and Karapinar (1997), Kabak and Dobson (2011)
Burukutu	Guinea corn	*Acetobacter* spp*., Candida* spp*., Enterobacter* spp*., Lactobacillus* spp*., S. cerevisiae, S. chavelieri, Lc. mesenteroides*	Consumed as an alcoholic beverage	West Africa	Adebo (2020), Bala et al. (2017)
Bushera	Sorghum	*Lactobacillus* spp*., Lactococcus* spp*., Leuconostoc* spp*., Enterococcus* spp*.,* and *Streptococcus* spp*.*	Consumed by all ages and used as a weaning food and a thirst-quenching drink in the households	Uganda	Achi and Asamudo (2019), Adebo (2020), Muyanja et al. (2003)
Chibuku	Maize and sorghum	*Lactobacillus* spp*.* and* S. cereviseae*	Consumed as alcoholic beverage	Zimbabwe	Adebo (2020), Mawonike et al. (2018)
Dolo	Red sorghum grains	*L. delbrueckii, L. fermentum, L. lactis, P. acidilactici, S. cerevisae*	Consumed as alcoholic beverage	Burkina Faso/Togo	Adebo (2020)
Dosa (fermented paste fried as pan-cake)	Rice and lentils	*Leuconostoc* spp*., mesenteroides* spp*., Streptococcus* spp*., Lactobacillus* spp*.*	Consumed during breakfast	India	Gupta and Abu-Ghannam (2011), Sawadogo-Lingani et al. (2021)
Enturire	Sorghum	*L. plantarum*, *S. cerevisae*, *Weissela confusa*	Consumed as alcoholic beverage	Uganda	Adebo (2020)
Gowe (sifanu)	Sorghum and maize flours	Lactic acid bacteria, such as *Lactobacillus* spp*.* and yeasts	Consumed as beverage by people living in urban areas	Benin	Achi and Asamudo (2019)
Humulur	Sorghum	*Bacillus* spp., *Lactobacillus* spp., yeasts	Used as a gruel	Sudan	Adams (1998), Adebo (2020)
Idli (steam cooked fermented paste)	Rice flour	*Lc. mesenteroides, S. faecalis, L. delbrueckii, L. fermenti, L. lactis,* and *P. cerevisiae*	Consumed during breakfast	India	Gupta and Abu-Ghannam (2011)
Ikigage	Sorghum	*Issatchenkia orientalis*, *L. buchneri, L. fermentum*, *Lactobacillus* spp., *S. cerevisiae*	Consumed as alcoholic beverage	Rwanda	Lyumugabe et al. (2010)
Injera	Teff	*C. guillermondii*, *Lactobacillus* spp, yeasts	Consumed as flat bread	Ethiopia	Achi and Asamudo (2019)
Khambir	Wheat	*L. brevis, L. plantarum, L. Casei, Bacillus,* mold, yeast, and lactic acid bacterial	Consumed as flat bread	Middle east countries	Tsafrakidou et al. (2020)
Kenkey	Corn	Lactic acid bacteria, *Enterobacter* spp*.* and yeasts	Consumed as cooked/steamed dough	Ghana	Achi and Asamudo (2019), Gupta and Abu-Ghannam (2011), Nout et al. (1995), Wesolowska et al. (2018)
Kisra	Sorghum flour	*C. intermedia*, C. *krusei*, *Debrayomyces hansenii, Enterococcus faecium*, *L. amylovorus*, *L. brevis*, *L. confusus*, *L. fermentum*, *Pichia kudriavzevii*	Consumed as flat bread	Sudah, south Africa	Achi and Asamudo (2019)
Kunun-zaki	Millet grains	*Lactobacillus plantarum, Lb. fermentum,* and* Lactococcus lactis*	Consumed as a refreshing drink, an appetizer, a food complement, and to quench thirst	Nigeria	Achi and Asamudo (2019)
Kwete	Sorghum, millet or maize	*Lactobacillus* and* Lactococcus*	Light brown in color, with a thick consistency and a sweet–sour taste	Uganda	Achi and Asamudo (2019)
Mahewu	Corn meal	*Lc. lactis*	Consumed as beverage, prepared from the maize porridge	Africa and some arabian arabian gulf	Achi and Asamudo (2019), Gupta and Abu-Ghannam (2011)
Mbege	Black/ white glutinous rice	*L. plantarum, Leuc. mesenteroides*, *S. cerevisiae, Schizosaccharomyces pombe*	Consumed as beverage	Tanzania	Achi and Asamudo (2019)
Merissa	Dates, millet and sorghum	*Saccharomyces* spp.	Consumed as alcoholic drink	Sudan	Achi and Asamudo (2019)
Obiolor	Sorghum and millet malts	lactic acid bacteria and yeasts	Consumed as beverage, served as functional foods Daily consumption and associated with a good health	Nigeria	Achi and Asamudo (2019)
Ogi	Corn	*L. acidophilus*, *L. agilis*, *L. cellobiosus*, *L. confusus*, *L. murinus*, *L. plantarum*	Popular breakfast gruel and a complementary food for children	Sub-Saharan Africa	Achi and Asamudo (2019), Gupta and Abu-Ghannam (2011)
Ori-ese	Sorghum	*Bacillus subtilis*, *C. tropicalis*, *L. acidophilus*, *L. fermentum*, *L. plantarum*, *Mucor* spp., *Pediococcus* spp., *Penicillium* spp., *S*. *pombe*	Consumed as porridge	Nigeria	Achi and Asamudo (2019)
Otika/Pito	Sorghum	*B. cereus, B. subtilis, C. krusei, C. tropicalis, Enterobacter clocae, L. brevis,L. fermentum*, *L. plantarum*, *Leuconostoc mesenteroides, S. cerevisae*	Consumed as alcoholic beverage	Nigeria	Achi and Asamudo (2019)
Pozol	Maize	*Lc. mesenteroides, L. plantarum, L. confusus, Lt. lactis and Lt. raffinolactis*	Used for making dough	Mexico	Gupta and Abu-Ghannam (2011)
Tarhana	Wheat flour	*L. delbrueckii and S. cerevisiae, Lc. lactis, Lc. diacetylactis, Lb. acidophilus, Lb. casei, and Leuconostoc cremoris*	Consumed as a snack	the Middle East and Turkey	Kabak and Dobson (2011)
Tchapalo	Sorghum	*L*. *brevis*, *L*. *cellobiosus*, *L*. *coprophilus*, *L*. *fermentum*, *L. hilgardii*, *L*. *plantarum*	Consumed as alcoholic beverage	Ivory Coast	Achi and Asamudo (2019)
Tchoukoutou	Sorghum and millet	*L. divergens, L. fermentum, L. fructivorans, S. cerevisae, S. pastorianus, Torulasposa delbrueckii*	Consumed as alcoholic beverage	Benin	Kayodé et al. (2007)
Tella	Wheat, maize, sorghum, and teff	*L. pastorianumi*, *S. cerevisae*	Consumed as beverage	Ethiopia	Wedajo Lemi (2020)
Ting	Sorghum	*L. casei*, *L. coryniformis*, *L. curvatus*, *L. fermentum, L. harbinensis*, *L. parabuchneri*, *L. plantarum*, *L. reuteri*, *L. rhamnosus*	Consumed as porridge	Botswana, South Africa	Sekwati-Monang et al. (2011)
Toddy	Palm trees	*Bacillus subtilis*	Used for sap from coconut	Sri Lanka, Nepal, Bangladesh	Gupta and Abu-Ghannam (2011)
Togwa	Sorghum, maize, millet and maize–sorghum	*Lactobacillus* spp*, P. pentosaceus, Weissella confusa, Issatchenkia orientalis, S. cerevisiae, C. pelliculosa,* and* C. tropicalis*	Consumed by the working people and also used as refreshment and a weaning food, with sweet taste and sour nonalcoholic beverage	Tanzania	Achi and Asamudo (2019)

B.: *Bacillus, L.*: *Lactobacillus*, P.: *Pediococcus, Lc.: Leuconostoc*, *H.: Hanseniaspora, K.: Kodamaea, E.: Enterococcus, P.: Pediococcus, T.: Tetragenococcus.* LAB: Lactic acid bacteria, Lt.: *Lactococcus*, C.: *Candida*

In several African nations, alcoholic and non-alcoholic beverages are made from fermented sorghum and millet (Achi and Asamudo 2019). They serve as the base grains for the non-alcoholic beverage bushera and the traditional alcoholic beverage muramba. *Lactococcus, Leuconostoc, Lactobacillus, Weissella,* and *Enterococcus* are responsible for bushera production (Muyanja et al. 2003). While Ethiopian customers are well-versed with barley meals and beverages, including Kunun-zaki, shorba, kinche, tihlo, shamet, chuko, beso, etc., is another millet-fermented beverage that is frequently enjoyed in Northern Nigeria. Lactic acid fermentation is a typical, simple, and affordable method for processing foods, including starch, to ferment cereal products. Cereal food products’ nutritional and organoleptic value is improved through lactic acid bacteria fermentation. The sensory qualities represent the first significant advancement. Bread, loaves, confectionary, pastes, noodles, gruels, semi-digested drinks, and supplemental meals for infants and children are produced by lactic acid fermentation of cereal substrate (Tsafrakidou et al. 2020).

### Fermentation of insects

In many civilizations worldwide, eating insects has long been a widespread practice (Anusha Siddiqui et al. 2022; Caparros Megido et al. 2016). Because of their better feed-conversion efficiency, reproductive potential, and environmental sustainability, insect-based products have gained favor as trendy new food items in addition to their excellent nutritional value (Niva and Vainio 2021). To enhance the sensory, nutritious, and shelf-life properties of new components and products and their availability and customer acceptance/receptivity, scientists and technologists have adopted both conventional and cutting-edge processing techniques (Caparros Megido et al. 2014; Mancini et al. 2019). With its ability to enhance fermented meals’ flavor, rheology, and texture and alter people’s perceptions of processed insect products, fermentation has attracted considerable study. Fermented sauces created with wax moth grasshoppers (*Locusta migratoria*) and larvae (*Galleria mellonella*) exhibited substantial acceptance for several sensory descriptors such as “sour”, “bitter”, “sweet”, and “umami”, when compared to a commercial fish sauce (Mouritsen et al. 2017). This behavior was due to increased fermentation-derived substances such as lactic acid, free amino acids, and fatty acids. There is much work to be done in order to harness the benefits of fermentation in order to enhance the sensory quality of insect-based food products and improve their availability in the marketplace. Moreover, this process may transform raw materials into biomass, fuels, and chemicals, all with a wide range of commercial uses. To treat edible insects, fermentation technology can potentially increase insect components' functioning and their use. The fermentation products of insects and bacteria are listed in Table 8.

**Table 8:** Fermented insects

Fermented insects	Insect source	Bacteria involved	Consumers attitude	References
Fermented cricket flour	Cricket	*Lactiplantibacillus plantarum*	Overally accepted remarked with “happiness”	Bartkiene et al. (2023)
Fermented *Tenebrio molitor* larvae	Larvae	*Cordyceps militaris* mycelia	Increased to healthy food products	Ha et al. (2022)
Fermented *Tenebrio molitor* larvae	larvae	*Aspergillus oryzae* and* Bacillus licheniformis*	Used as seasoning sauces	Cho et al. (2018)
Fermented silkworm larvae	Larvae	*Aspergillus kawachii*	Consumed as a food and traditional medicine	Cho et al. (2019)
Fermented yellow mealworm larvae	Larvae	*Pediococcus acidilactici, Lactobacillus curvatus,* and* Staphylococcus xylosus*	No consumer study observed, but expected to have shelf-life extension	Borremans et al. (2018)
Fermented mealworms	Larvae	*Pediococcus acidilactici, Lactobacillus curvatus,* and* Staphylococcus xylosus*	Used to extend the shelf life of the insect food	De Smet et al. (2019)

Compared to the fermentation of the previously mentioned items, the fermentation of insect feeds is relatively new. Cho et al. (2019) used an *Aspergillus kawachii* solid-state fermentation to treat the flour made by mulberry silkworm larvae (*Bombyx mori*). The scientists examined how fermented silkworms produced free amino acids, fatty acids, minerals, and alcoholic chemicals. In a study by Jang et al. (2018), several microorganisms were used to ferment yellow mealworm larvae (*Tenebrio molitor*, *Lactobacillus plantarum, Lactobacillus gasseri, Aspergillus kawachii, Saccharomyces cerevisiae,* and *Bacillus subtilis*). Following fermentation with each strain separately, extracts of water, ethanol, and methanol were made and used to evaluate the biological characteristics of the products.

According to the facts above, fermentation is a workable alternative to other methods of preparing active insect metabolites. These naturally occurring substances, produced by fermentation, might be utilized as components in functional foods and nutraceuticals. Due to the bioactive substances insects have produced through fermentation, they will one day provide consumers with health benefits. However, further study is needed to benefit from their future uses and numerous health benefits in the future. Indeed, fermentation offers a unique method of obtaining active compounds from insects that may be used in a variety of applications. These compounds can be used to produce medicines, vitamins, and cosmetics, among other things. They can also be used to create sustainable food products, such as protein bars, that can provide essential nutrients to those with limited access to a balanced diet.

### Fermentation of seaweed

Seaweed fermentation has been previously observed in food or medicine (Gullón et al. 2020, 2021; Wendin and Undeland 2020). The demand for seaweed in the Asian market, which has resulted in sharp increases in aquaculture yields, has been a significant factor in the continuous rise in seaweed consumption over the past few decades. Global seaweed harvesting was 29 million tonnes in 2016. Their primary uses were synthesizing hydrocolloids for food and pharmaceuticals, animal feed, and human consumption (Zhang et al. 2022). While early investigations indicated excellent yields, seaweeds are particularly ideal for this purpose because their development systems do not compete with crops and do not require fresh water.

**Fig. 2:**
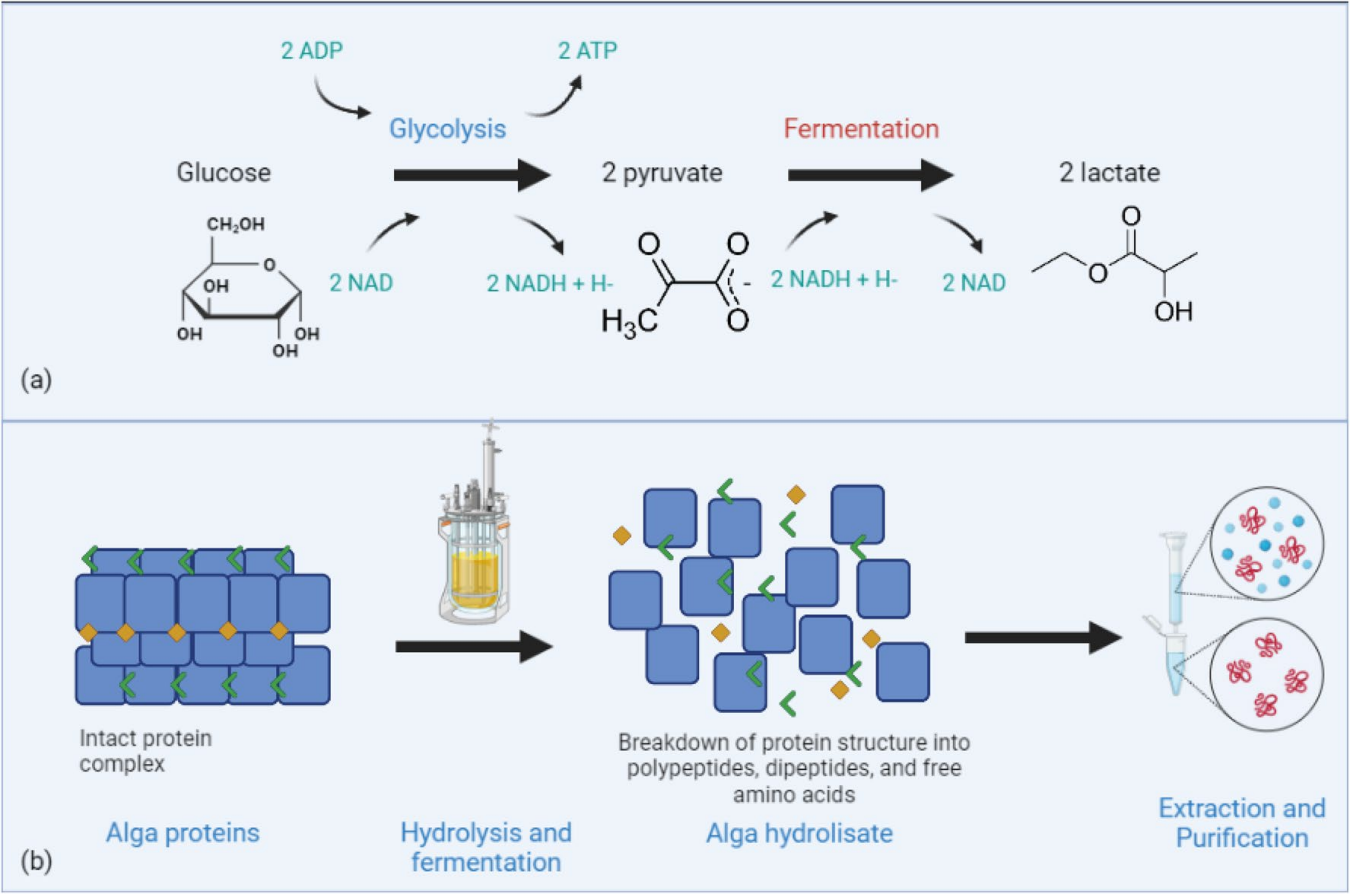
**a** Chemical fermentation path of the seaweeds, and **b** physical appearance of the fermentation of process in seaweeds. “Created with BioRender.com”

Seaweed fermentation products are most pertinent because the primary structural polysaccharides undergo hydrolysis, producing a high quantity of glucose (Monteiro et al. 2021). Laminarin, alginate, and fucoidan are found in brown seaweeds, agar and carrageenans in red seaweeds, starch and ulvan in green seaweeds, and laminarin, alginate, and fucoidan are found in brown seaweeds (Milinovic et al. 2021; Pérez-Alva et al. 2022). Brown seaweeds provide extra fermentable sugars in the form of mannitol and glucuronic acid that, provided mannitol-fermented cultures have been used, can further enrich the fermentable mash. These sugars and the hydrolyzed polysaccharides undergo glycolysis to produce pyruvate, which is subsequently fermented to produce either lactic acid or ethanol and CO_2_, as shown in Fig. 2. Most microbial cultures cannot use several seaweed sugars, including mannuronic and uronic acids, fucose, rhamnose, and xylose, making ethanol fermentation of seaweed difficult, so genetically modified cultures have been created to convert seaweed sugars well (Monteiro et al. 2021). Seaweed has an intact protein complex, hydrolyzed after fermentation. The fermentation also physically disrupts seaweed cells and consequently breakdown the protein–phenolic blends to release the Fermentation of various seaweeds depending on the sugar content in seaweeds to select the bacteria involved in the fermentation process, shown in Table 9. Seaweed *Gracilaria verrucosa* can be fermented with *Hortaea werneckii, Lactobacillus* spp*.,* and S*taphylococcus*. The fermentation of seaweeds increased antioxidant and antimicrobial activity (Fatmawati et al. 2022). The increase in antimicrobial activity implicated the protection of seaweeds from pathogenic and spoilage bacteria and improved the seaweeds' nutritional value by reducing the insoluble and indigestible fractions (Maiorano et al. 2022). Figure 2 shows the fermentation process. The fermentation process activates the seaweeds' antimicrobial activity which helps to protect them from bacteria and other contaminants. This process also aids in the breakdown of indigestible components, making the seaweeds more bioavailable and easier to digest. This makes them ideal for use in a variety of applications, such as biofuels, nutritional supplements, and pharmaceuticals.

**Table 9:** Fermented seaweed

Seaweed	Bacteria involved	Consumers attitude	References
Fermented *Gracilaria verrrucosa*	Marine yeast *Hortaea werneckii*	No consumer concern was observed, the fermented seaweeds had increasing antibacterial and antioxidant activity	Fatmawati et al. (2022)
Fermented *Gracilaria gracilis*	*Lactobacillus sakei, Staphylococcus carnosus* and* Staphylococcus xylosus*	Improving the nutritional value of the seaweeds	Maiorano et al. (2022)
Edible red seaweed* Bangia fusco-purpurea*	*Lactobacillus delbrueckii* and* Lactobacillus plantarum*	Consumed as special dietary food to improve hyperlipidemia and obesity	Li et al. (2023)
*Kappaphycus* spp	*Aspergillus oryzae*	As a food ingredient to improve the nutritional, taste and textural properties of food products	Norakma et al. (2022)
Green seaweed *Ulva* spp.	*Paradendryphiella salina* (marine fungus)	As a promising source of functional and sustainable ingredients for food	Landeta-Salgado et al. (2021)
Edible Irish brown seaweeds *Himanthalia elongata, Laminaria digitata* and* Laminaria saccharina*	*Lactobacillus plantarum*	As functional foods	Gupta et al. (2011)

Furthermore, bacteria involving the fermentation process can also utilize fungi found in the marine area. Landeta-Salgado et al. (2021) reported the fermentation of green seaweeds *Ulva* spp*.* hydrolyzed by the marine fungi *Paradendryphiella salina*. The results showed an increase in yield, protein, and amino acids. However, the research on the fermentation of seaweeds for functional foods still needs to be completed. Further analysis of fermented seaweeds’ effects on food processing is essential.

### Coffee fermentation

**Fig. 3:**
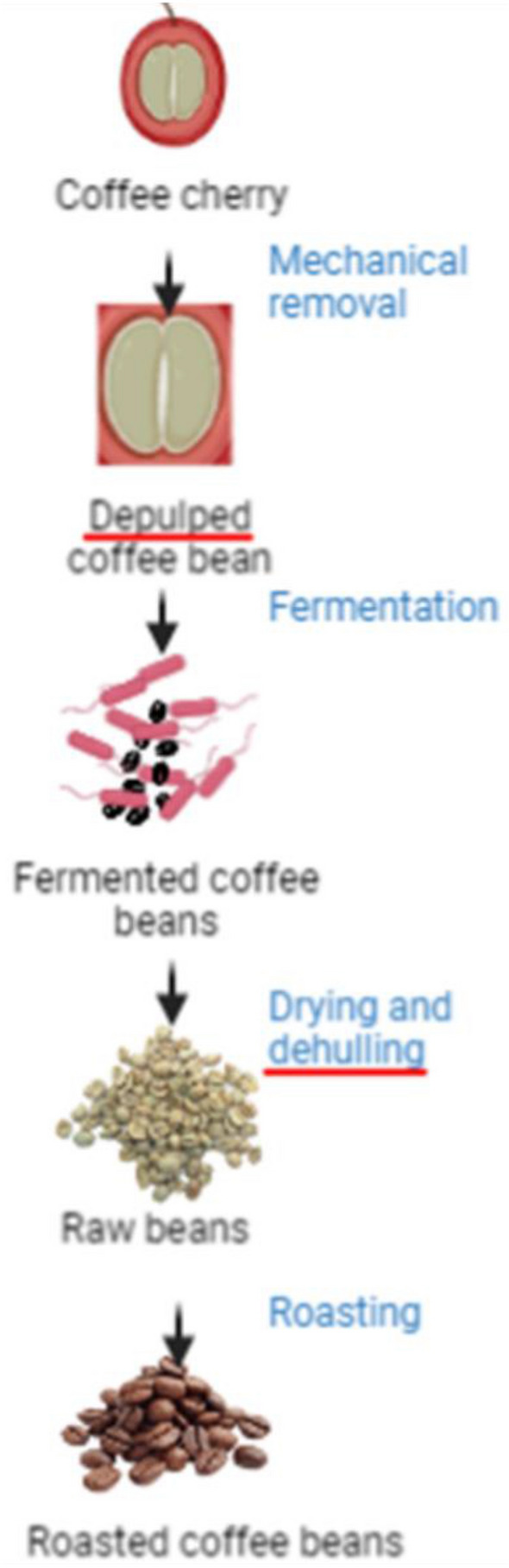
Coffee fermentation from wet processing. Created with BioRender.com

Coffee is a widely consumed beverage prepared from coffee beans. Despite fermentation being necessary to remove the mucilage layer, subsequent heat-intensive processes (roasting and brewing) produce a drink that is close, as shown in Fig. 3. During coffee fermentation, parchment coffee's mucilage must be removed. Coffee mucilage contains starch, cellulose, and pectin. The mucilage may make it difficult to dry coffee beans and, in rare cases, may also promote mold growth, lowering the final coffee quality (Haile and Kang 2019a). Spontaneous fermentation is often employed since it is explicitly done to remove mucilage (Haile and Kang 2019b).

Furthermore, coffee beans already contain all the ingredients needed to produce coffee flavor and fragrance during roasting (Joët et al. 2010). Yet, fermentation can broaden the variety of chemicals that give coffee flavor and fragrance, including more than 700 volatile and nonvolatile chemicals. According to reports, yeast, LAB, and Enterobacteriaceae are predominantly responsible for wet fermentation, whereas acetic acid bacteria and Pichia yeasts are responsible for dry fermentation (Lavefve et al. 2019), as shown in Table 6. Since the mid-1900s, numerous microorganisms have been isolated from wet processing fermentation. Since aromatic chemicals are created when the mucilage layer in wet processing is removed, wet-processed coffee has better scent attributes than dry-processed coffee (Haile and Kang 2019a). Current research on coffee fermentation during dry, semi-dry, and wet processing focuses on using aromatic yeasts to create flavor (De Melo Pereira et al. 2015; Evangelista et al. 2014). A wide range of microbial species (Table 10) are present during coffee fermentation; however, only a small number of these native microorganisms were chosen because of their potential effects on the coffee’s flavor and fragrance. Fermentation should be regulated to achieve this favorable outcome. The choice of suitable microorganisms that positively impact coffee flavor and fragrance during fermentation is crucial. Thus, it is important to properly regulate fermentation in order to achieve desired flavor and aroma in coffee. The choice of the right microorganisms is important because it is these microorganisms that produce the compounds responsible for the flavor and aroma of coffee. Therefore, by regulating the fermentation process, the desired flavor and aroma can be achieved.

**Table 10:** Fermentation of coffee

Fermented coffee	Bacteria involved	Consumers attitude	References
Wet fermented coffee	LAB, *Enterobacteriaceae*, and Yeast	Consumed for refreshment drink	De Melo Pereira et al. (2015), Lavefve et al. (2019)
Dry fermented coffee	Acetic acid bacteria and Pichia yeasts	Consumed for refreshment drink	Evangelista et al. (2014), Lavefve et al. (2019)

## Precision fermentation

**Fig. 4:**
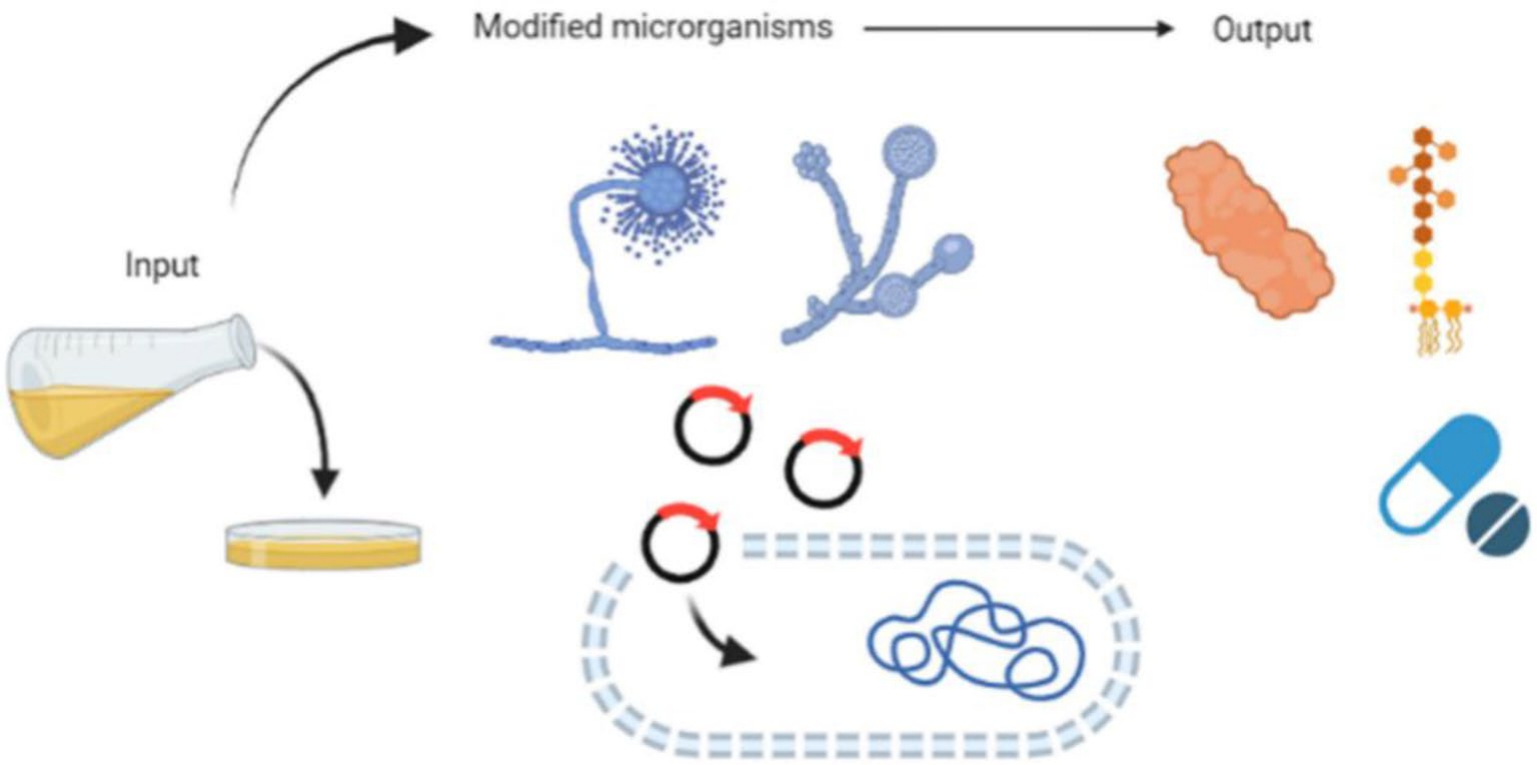
Precision fermentation. “Created with BioRender.com”

Producing edible microbes has the potential to bypass many of the environmental constraints of food production and reduce its environmental footprint at a time when climate change threatens the global food production system (Linder 2019). The use of fermentation has been a useful method of food preservation in the past. The microbial populations involved made the resulting products typical concerning where processing occurred (Campbell-Platt 1994). Precision fermentation uses synthetic biology, especially genetic engineering, to insert specific genes into the DNA backbone of single-celled organisms and microorganisms to produce desired fermentation properties and products (Augustin et al. 2023). One way to reduce by-product formation is to create synthetic cellular factories in which all available resources are diverted to produce the compounds needed and nothing more. Known as precision fermentation or synthetic biology, the technique is now being touted as a potential alternative to traditional fermentation (Fig. 4). The focus is designing optimized metabolic pathways and assembling the genes involved in the microbial chassis. In order to analyze and characterize microbial genomes and metabolic functions, this technology relies heavily on artificial intelligence, bioinformatics, systems biology, and computational biology (Teng et al. 2021).

Recent advances based on genomics and synthetic biology include precision fermentation and biomass fermentation to produce specific compounds for food and chemical industrial or pharmaceutical purposes (Teng et al. 2021). A variety of vitamins are found in microbial biomass, including biotin, folic acid, niacin, pantothenic acid, pyridoxine, riboflavin, and thiamine (Ritala et al. 2017). Rapid advances in precision biology enable microbial programming to produce complex organic molecules (Augustin et al. 2023). Sugars, alcohols, organic acids, and hydrocarbons are simple organic feedstocks that can be used to culture microorganisms. In the field of carbohydrates, precision fermentation for the production of oligosaccharides has received the most attention. Several patents describe the construction of genetically engineered microbial cells to enhance oligosaccharide production. The primary targets are human milk and its associated oligosaccharides applied to infant formula and supplements. These oligosaccharides provide non-nutritional biological properties to infants, such as promoting gut health and the growth of beneficial microflora in the gut (Ambrogi et al. 2023; Barile and Rastall 2013). Microbial biomass producers often prefer low-cost organic by-products from the food industry, such as molasses, vegetable starch, and whey (Linder 2019). Microbial biomass production costs are also strongly influenced by choice of organic substrate for microbial growth (often called feedstock) (Linder 2019). The use of fermentation has been a useful method of food preservation in the past. the microbial populations involved made the resulting products typical concerning where processing occurred (Campbell-Platt 1994).

Large-scale production of fermented food products involves the use of starter cultures. defined aliquots of selected microbial cultures are added to the substrate to be fermented. depending on the selected starter culture, the fermentation process can be directed. for example, lactic acid bacteria (LAB) ferment lactose present in the substrate by lowering the pH; they inhibit the growth of undesirable bacteria but also contribute to the flavor and texture of the food (Parente et al. 2017). There are still inherent risks, especially when using traditional or spontaneous fermentation. Pathogenic microorganisms or harmful metabolites can spoil the final product and pose a health risk to consumers. Therefore, applying genomic analysis will help improve safety through the early detection of harmful microorganisms. Finally, combining genomics and synthetic biology to design desirable traits rationally holds promise for using non-food biomass to create new foods that are safe, healthy, and appealing to consumers (Teng et al. 2021). Genomic studies of food microbiomes have made great strides as rapid advances in sequencing technologies have greatly reduced sequencing costs and led to an increasing number of genomes published in public databases. Metagenomic analysis using HTS helps to reveal metabolic functions and parameters that affect fermentation processes, such as B. Substrate consumption, enzyme production, or metabolic output. Metabolic modeling combined with flux balance analysis to simulate microbial growth and metabolite production in response to changes in the culture environment (Alkema et al. 2016). Altogether, metabolic analysis and modeling would not only help to improve yield, taste, or texture in industrial-scale fermentation based on starter cultures but could also benefit smaller, artisanal producers to avoid contamination or spoilage by suppressing the growth of unwanted microbes or metabolic functions(Teng et al. 2021) By pushing the limits of precision fermentation; we can envision future food production systems in which fermentation plays a central role in producing a variety of food products.

In precision fermentation, microorganisms are programmed using synthetic biology techniques to produce food and pharmaceutical ingredients as cell factories (Pham 2018). Selection of recombinant host microorganisms and strain engineering is the first task to determine the possibility of constructing a microorganism that expresses and produces target molecules in sufficient quantities using appropriate fermentation conditions to increase production efficiency. Microbial hosts are preferred because they are easily manipulated genetically, and standardized fermentation equipment can be used. For food applications, strain engineering often utilizes benign bacteria (e.g., *Bacillus* spp.) and yeasts because microorganisms that are generally considered safe (GRAS) or harmless are preferred for food applications and so strain engineering often utilizes benign bacteria (such as *Bacillus* spp.), yeasts (such as *Saccharomyces cerevisiae, Pichia pastoris* (now *Komagataella phaffii*), *Kluyveromyces* spp.), or filamentous fungi (such as *Trichoderma* spp., notably the popular *T. reesei* strains) (Chai et al. 2022a, b).

Innovation can occur through novel species and strains or by leveraging genetic engineering and synthetic biology techniques to optimize the yield of desired products, for example, by improving expression, secretion, substrate conversion, and product titer. The untapped potential of natural microbial biodiversity to provide efficient microbial cell factories for novel and safe fermented foods is revealed by “omics” tools and recent advances in synthetic biology. These tools can be utilized to develop products that match desired characteristics and precisely control the fermentation process rather than randomly (Teng et al. 2021). Precision fermentation is easily suited for producing selected proteins, lipids, and carbohydrates due to its ability to produce molecules that mimic comparable compositions derived from conventional agriculture. For example, the oleaginous red yeast *Rhodosporidium toruloideshas* has been genetically engineered to improve the natural synthesis of lipids, carotenoids, and novel compounds of industrial importance. A thorough understanding of the metabolism of microbial communities can also facilitate the development of new substrates, especially by-products and wastes of the food industry, for the production of value-added products. The research team has demonstrated the value of fermenting food waste with the probiotic *Bacillus subtilis* to create new foods with higher nutritional value (Mok et al. 2019). A more sustainable strategy is biomass fermentation for protein production, based on the ability of microorganisms to reproduce rapidly under optimal conditions and produce favorably high protein contents exceeding 50% dry weight. Examples of such products are Marmite made from yeast extract, fermented bean paste, and fungal protein from the filamentous fungus *Fusarium venenatum*, which has recently been used as a raw material for artificial meat (Berka et al. 2004).

From a genomics point of view, single-cell protein production could be comparatively easily explored by high-throughput strain screening, adaptation, and engineering to engineering microbial strains and cell factories for protein production (Teng et al. 2021) Fungi are eukaryotic and saprophytic microorganisms with solid environmental adaptability, making them suitable microbial hosts for precision fermentation. The natural tendency of many fungal species to accumulate high levels of commercially valuable food compounds (organic acids, carotenoids, polyketides, and fungal pigments) makes them conveniently efficient hosts for the industrial-scale production of these products. From a metabolic engineering perspective, a key strength of using fungi compared to bacteria is that their eukaryotic nature makes them tolerant and able to functionally express heterologous eukaryotic proteins and enzymes, achieving proper protein folding and post-translational modifications (Lyu et al. 2019). Many species of fungi are involved in food production, such as *Saccharomyces cerevisiae* whose genome has been sequenced and allowed to produce vanillin and other aromatic compounds (Nxumalo and Thimiri Govinda Raj 2020). Another fungus used in precision fermentation is *Yarrowia lipolytica*, a ubiquitous oleaginous fungus that tolerates wide variations in pH and salinity (Miller and Alper 2019). it has the unique metabolic ability to degrade hydrophobic and lipophilic substrates and accumulate more than 40 percent lipid by dry cell weight. It is an excellent source of lipid derivatives or fatty acids (Bilal et al. 2021). Other fungi, besides those mentioned above, can also produce valuable compounds. *Kluyveromyces lactisin* particular, is an established commercial lactase producer, and its commercial production of recombinant bovine chymosin could be considered a pioneering success in precision fermentation. Fungi play a significant role in traditional fermentations, including beer, wine, bread, cheese, sauces, vegetables, and meat.

In contrast to precision fermentation, most commercial traditional food fermentations are artisanal, natural, and largely undefined, as exemplified by the imprecise method of backing. In recent years, however, precise methods are increasingly being explored and applied to traditional fermentations to speed up the process, increase product yields, improve food quality, safety, nutrition, and flavor profiles, and reduce process costs. Such methods include high-throughput screening strategies, CRISPR-Cas9 genome editing tools, and multi-omics (Chai et al. 2022a, b).

## Valorization of food waste by fermentation

Food waste (FW) comprises complex carbohydrates, proteins, lipids, organic acids, enzymes, and nutraceuticals (Carmona-Cabello et al. 2018; O’Connor et al. 2021; Ravindran and Jaiswal 2016). Although its definition has been widely debated, according to the Food and Agriculture Organization (FAO), it is defined as the “total occurred qualitative and quantitative food losses during the supply chain process, which happens at the different stages like production, post-harvesting and processing”. FW is usually considered a non-dangerous waste, except for animal-derived waste strictly controlled by the European regulation (EC) No 1069/2009. FW is becoming a growing and vital problem locally and globally. In fact, according to the FAO, one-third of all food production is lost or wasted globally every year. FW is traditionally disposed of in landfills or incinerated for energy production (Melikoglu et al. 2013). The disposal of FW in landfills is related to several adverse environmental effects (Pires et al. 2021).

In addition, FW is responsible for more than 20% of the total global production of greenhouse gases (GHC), including methane (CH_4_), nitrous oxide (N_2_O), and carbon dioxide (CO_2_) (Munesue et al. 2015). For this reason, the prevention of its products, together with its valorization, is of crucial importance. Indeed, due to the growing public awareness of the indiscriminate disposal of FW and its harmful ecological impact, there is an increasing interest in the recycling and bioconversion of FW, and for this, FW valorization is becoming an expanding industry. Valorization of FW refers to the processes for converting food waste materials into a range of more valuable products. The recycling and the bioconversion of FW significant opportunities to support sustainable development (Capson-Tojo et al. 2016). Fermentation is one of the oldest approaches used for product transformation into value-added products using microorganisms. In fact, by converting these by-products through the microbial fermentation process, different value-added products can be produced, including feed and food additives, single-cell protein (SPC), biofertilizers, bioplastics, chemicals, fuels, food grade pigments and nutraceuticals (Lin et al. 2019). Moreover, FW valorization will bring economic, environmental, and social benefits through, in addition to the manufacture of value-added products, the mitigation of environmental pollution, and overcoming the issues related to odor and the spread of pathogens (Bilo et al. 2018).

**Fig. 5:**
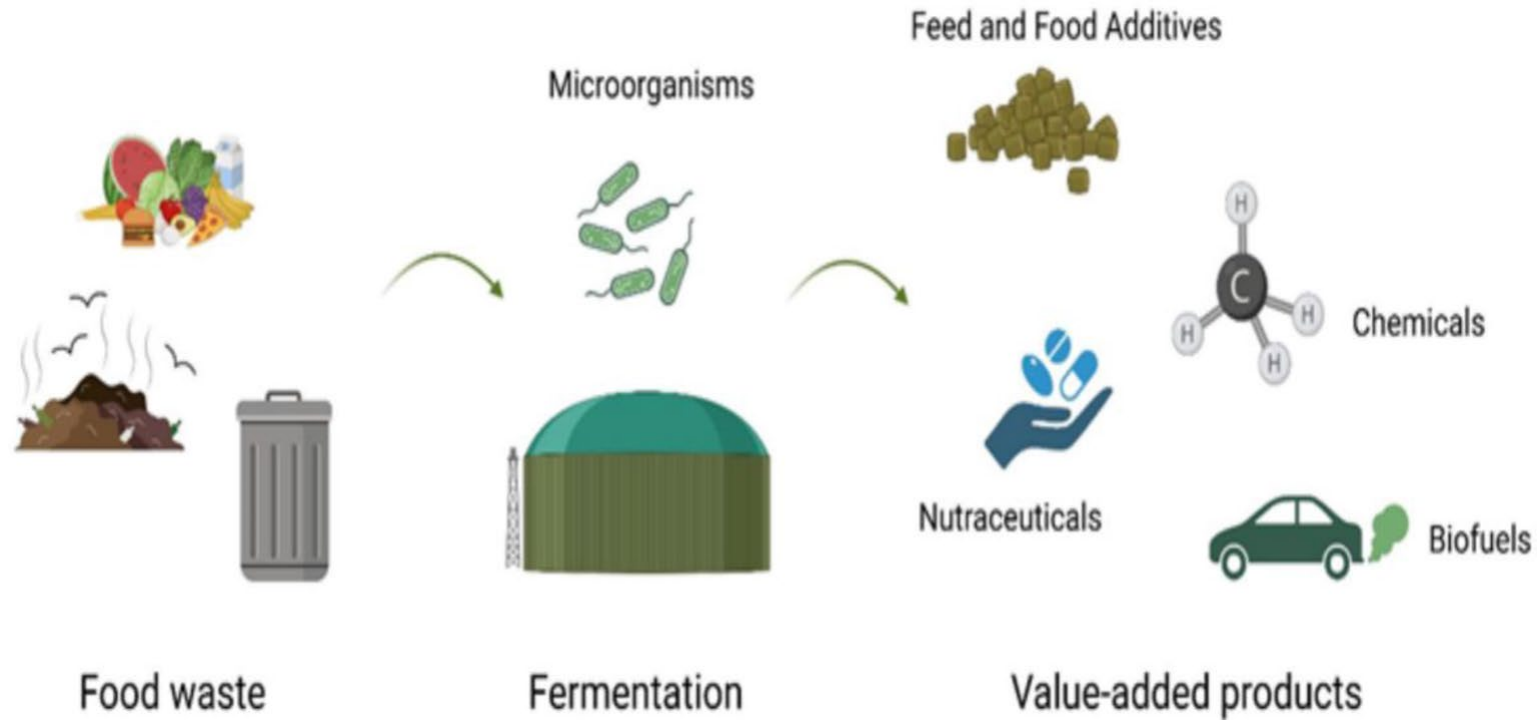
Valorization of food waste by fermentation. “Created with BioRender.com”

Intending to valorize food waste, several promising technologies using acidogenic fermentation (Fig. 5) with anaerobic microbial communities are taking hold to generate different value-added products from biowastes (Ortiz-Sanchez et al. 2023). These techniques are often alternative or supplementary to more conventional ones and employ anaerobic digestion (Palacios et al. 2017). The transformation of bioproducts via natural processes is one of the significant advantages. The resulting products are safe and healthy for human consumption (Pires et al. 2021). Several are the products that can be recovered from FW. For instance, the carbohydrates in the FW can be fermented to produce lactic acid, ethanol, volatile fatty acids (carboxylic acids with 1 to 4 carbon atoms, VFA), or hydrogen (De Groof et al. 2021; Im et al. 2020; Tang et al. 2017; Zhang et al. 2021) and these products can be then extracted to serve as renewable commodity chemicals or liquid fuels (Kannengiesser et al. 2016). Moreover, to mitigate the negative impact, an enhanced approach to the waste management of the fruits and vegetables processing industry is a critical step in the transition to the bioeconomy. It is now well known that agro-industrial activity creates multiple and different types of waste, which are susceptible to being spontaneously fermented by the microbiota present, of course, in these by-products (Sabater et al. 2020; Valenti et al. 2020).

Many studies dealing with the fermentation of fruits and vegetable by-products as an alternative way of valorizing food waste using different microorganisms have been reported in the literature (Lu et al. 2022). Several examples are reported in the literature based on fermentation-based valorization strategies, including the anaerobic digestion of organic feedstock, date palm waste, cocoa by-products, and sourdough to produce lactic acid as an alternative for raw material, enzymes, polysaccharides, beverages, and nutraceuticals (Vasquez et al. 2019). The bacteria commonly used in controlled food manufacture are *Streptococcus thermophilus*, *Lactococcus lactis*, *Leuconostoc* spp., and *Lactobacillus* spp., for dairy products and the genera *Pediococcus,* *Oenococcus*, and *Weissella* play a pivotal role in plant-based fermented products (Bachtarzi et al. 2019). These valorization strategies using these bacteria, which we can also refer to as lactic acid bacteria (LAB), counted the production of lactic acid that could be replenished in the food chain, as well as improving the digestibility of proteins and the sensory properties of these plant by-products that could be used for food ingredients. However, fermentation strategies and bioconversion processes have been described to increase digestibility, enhance nutritional value and decrease the levels of antinutritional factors in these substrates, also employing other bacterial species, yeast, and molds (Yue et al. 2014). Therefore, the fermentation of agro-feed residues by LAB, alone or in combination with other microorganisms, paves the way for developing new sustainable circular economy strategies. In addition to fermentation strategies using LAB, other fermentative bacteria are reported to have been applied to valorize vegetable by-products (Table 11) and other vegetable sources, including different *Clostridium* and *Bacillus* bacterial species. Most of these applications focused on producing functional ingredients, such as lactic acid, poly-γ-glutamic acid, bioactive peptides to be reintegrated into the food chain, and other compounds like glycosidases or caproate of industrial interest. Regarding the genus *Bacillus*, *Bacillus coagulans*, *Bacillus amyloliquefaciens*, *Bacillus licheniformis* and *B. subtilis* have been used, alone or in combination with other bacterial species and with fungi, to ferment products derived from rice, soy, oak, fruit, sorghum (Tropea 2022). On the other hand, it should be noted that the Clostridium bacterial species is mainly used in the fermentation fruit waste. For example, in this regard, a successful fermentation strategy has been reported using both *Clostridium cellulovorans* and *Clostridium beijerinckii* strains to ferment mandarin orange waste. In this study, it has been demonstrated that, although normally, D-limonene included in citrus fruits inhibits yeast activity and makes ethanolic fermentation difficult; however, the physiological concentration of D-limonene does not inhibit the growth of the two Clostridium strains. Thus, starting from the isopropanol-butanol-ethanol fermenting ability of *C. beijerinckii* and the cellulosic biomass-degrading capacity of *C. cellulovorans* allows biofuels to be produced from this particular specific fruit waste (Yalemtesfa and Tenkegna 2010). Moreover, it has been highlighted the possibility of using vegetable and fruit waste to generate bioenergy in the form of biofuel. Fruit wastes, in particular, were used in the production of bioethanol. Instead, vegetable wastes, high in cellulose, hemicelluloses, and lignin, were employed to produce second-generation bioethanol (Thi et al. 2016). Moreover, Soya by-products were mostly subjected to solid-state fermentation at 30–47 °C using *Aspergillus niger* and *Bacillus* species or yeast employing lower temperatures (20–28 °C) (Wang et al. 2019). Instead, Barley bran and brewing waste were mostly inoculated with *Aspergillus Trichoderma* and LAB species. Another example of the use of fermentation to valorize FW is reported by Brancoli et al. (2021). The authors reported a solid-state fermentation process carried out by the edible fungus Neurospora intermedia using bread waste as feedstock for producing a protein-rich food product. In this research, which can contribute to highlighting how it is possible to manage wasted bread more sustainably, it has been proposed that solid-state fermentation could be used to recover the otherwise discarded surplus bread (Brancoli et al. 2021; Tropea 2022). Another opportunity is the possibility of using food industry waste as animal feed. This possibility appears to be very interesting, as it would bring both environmental and public benefits besides reducing animal production costs. As reported by Tropea et al. (Tropea et al. 2021), among the microbial cultures used in the biotechnological methods to recover food waste, lactic bacteria have several advantages over other bacterial species, especially in animal/fish processing wastes. They are, in fact, generally recognized as safe (GRAS).

**Table 11:** Food waste valorization

Food waste	Microorganism	Fermentation process	References
Sweet potato waste, banana skin, orange peel and mango waste	*Saccharomyces* sp*., Saccharomyces cerevisiae, Candida tropicalis, Lactobacillus acidophilus*	Solid fermentation; liquid fermentation;	Dulf et al. (2016)
Pineapple waste, banana waste	*Saccharomyces cerevisiae*	Solid fermentation	Verotta et al. (2018)
Rice bran, Brewery waste	*Lactobacillus delbuieckii* spp., *Bacillus licheniformis*, *Aspergillus niger*	Liquid fermentation, solid-state fermentation	Mathias et al. (2017)
Dairy waste (whey)	*Cryptococcus albidus* sp. Aerius	Liquid fermentation	Nemeth and Kaleta (2015)
Potato peel	*Xanthomonas citri*	Solid-state fermentation	Vidhyalakshmi et al. (2012)
Orange peel	*Chaetomium* spp. (KC-06) and *Aspergillus niger*	Solid-state fermentation	Yalemtesfa and Tenkegna (2010)
Peanut waste, pomaces and brandy distillery wastes, pomegranate wastes	*Rhizopus oligosporus: Aspergillus awamori, Rhizopus oryzae; Aspergillus niger* and *Rhizopus oligosporus, Punica granatum*	Solid-state fermentation	Sadh et al. (2018)
Apricot pomace, apple pomace	*Aspergillus awamori, Aspergillus niger* (ATCC-6275) and *Rhizopus oligosporus* (ATCC-22959); *Phanerocheate chrysosporium*	Solid-state fermentation	Sadh et al. (2018)
Olive pomace, bakery waste	*Yamadazyma guilliermondii*, *Yarrowia lipolytica*; *Xantophylomyces dendrorhous*, *Sporidiobolus salmonicolor*; *Monascus purpureus*	Solid-state fermentation	Dursun and Dalgıç (2016)
Tomato waste	*Aspergillus niger*	Solid-state fermentation	Jamal and Akbar, (2016)
Cotton seed meal, soy bean powder and wheat bran	*Streptomyces fradiae* NCIM 2418	Solid-state fermentation	Dulf et al. (2016)
Pulp and paper solid waste	*Rhizopus oryzae* 1526	Solid-state fermentation	Das et al. (2016)
Rice bran	*Aspergillus oryzae* and *Rhizopus oryzae*	Solid-state fermentation	Das et al. (2016)

Moreover, it has been shown that the products obtained upon the fermentation with *Lactobacillus* are also reported to have other beneficial effects on aquatic animal intestines, such as antimicrobial and antioxidative properties (Hoseinifar et al. 2014). Fermented fish waste appears as a liquid product, obtained from the liquefaction of tissues carried out by the enzymes already present in the fish and expedited by an acid pH (Tropea et al. 2021). It has been observed that these fish-derived products can rapidly adapt to the intestines of both aquatic and domestic animals, thus making it possible for them to be used in probiotic aquaculture feeds. For instance, studies are reported in the literature in which fish by-products (non-edible parts such as head, viscera, skin, and bones) of *Dicentrarchus labrax* are fermented by the microorganisms *S. cerevisiae* strains and *Lactobacillus reuteri* strains (Tropea et al. 2021). It has also been shown a fermentation process using non-sterilized fish wastes, supplemented with lemon peel as a filler and prebiotic source, carried on by two combined starter cultures of *Saccharomyces cerevisiae* and *Lactobacillus reuteri*. In this process, fish waster was bio-converted into a high protein content supplement used for aquaculture feeds. The final fermented product was found to be poor in spoilage microorganisms and rich in healthy microorganisms by displaying a lipid and protein content that makes it suitable for aquaculture feed. These results encourage fish waste and lemon peel conversion into animal feed (Tropea et al. 2021). Munesue et al. (2015) showed another use of food waste valorization through fermentation. Their study used pomaces, i.e., the waste generated from pressing fruits and olives to obtain juices and olive oil, as a feed supplement for animal production. The authors reported that the food obtained from animals fed with fermented pomaces was free from negative effects and with an improvement in nutritional quality.

Fermentation processes for valorizing food waste can also be an attractive opportunity to obtain new value-added products in other fields besides food, such as the cosmetic and pharmaceutical fields. A study conducted by Ferracane et al. (2021) was intended to evaluate the production and characteristics of soaps made from non-edible fermented olive oil (NEFOO soap) by assessing the pH, color, and solubility. Moreover, the glucan and pectin contents found in the green husks of walnuts grown in two different soil and climate areas of Southern Italy were also evaluated for their potential use after fermentation in food, cosmetic, and pharmaceuticals fields in a study carried out by La Torre et al. (La Torre et al. 2020). To date, few studies focus on the possibility of producing biogas from fermented FW. This biogas could then be used for heat or electricity production. In particular, there are several biotechnological processes, like one or two-stage fermentation, dark fermentation combined with aerobic digestion, and photo fermentation combined with aerobic digestion, which can be used to convert the plenty of organic fraction present in the FW to hydrogen. A study has shown that the higher the carbohydrate content of food waste, the better it will be valorized and converted into H_2_. Studies also show that food waste is suitable for methane production thanks to its physical and chemical characteristics (Thi et al. 2016). Finally, Panyawoot et al. (2022) conducted a study to evaluate the effects of the feed obtained via fermentation on final consumers. In this study, the authors assessed the impact of fermented discarded durian peel with *Lactobacillus casei*, cellulase, and molasses alone or combined in total mixed rations on feed utilization, digestibility, ruminal fermentation, and nitrogen utilization in growing crossbreed Thai Native-Anglo-Nubians goats. The research highlighted that the discarded durian peel fermented with molasses and *L. casei* had a much more excellent digestibility and propionate concentration. On the other hand, this product led to less methane and urinary nitrogen production.

## A future perspective on fermentation

Fermentation methods have produced many products for many years, including chemicals, materials, food, and medicines. In general, and excluding medicines, industrials fermentations are less competitive compared to the chemical industries or agriculture. More and more products can be obtained by fermentation through a safe, green, and sustainable process. However, for this technology to be increasingly competitive in the future, some issues must be solved. Some of them are freshwater shortage, heavy energy consumption, microbial contamination, the complexity of sterile operations, poor oxygen utilization in the cultures, food-related ingredients as substrates et al. For these reasons, future fermentation processes should be more effective and better from the point of view of the issues just mentioned (Chen and Jiang 2018). First, new fermentation methodologies should avoid the problems of bacterial contamination. Indeed, the microorganisms currently used are very susceptible to contamination by other microorganisms. For example, bacterial strains commonly used for fermentation methods, such as *E. coli* and *Bacillus* spp., *Corynebacterium glutamicum*, and *Pseudomonas* spp., and also yeast is grown in mild conditions, which also allow the growth of most microorganisms present in the air, in the water or the soil (Chen and Jiang 2018). For this, production facilities must be sterilized entirely to prevent microbiological contamination. This makes processes extremely complex, which also impacts the cost of production, which becomes higher. Therefore, the use of more resistant microorganisms and the implementation of measures to prevent contamination are two main requirements for the future improvement of fermentation processes. A study conducted on two bacteria *Halomonas* spp. and *Xerophiles* demonstrated that using these bacterial strains could answer the challenge of bacterial contamination. These microorganisms, extremophilic or unique substrate-selected bacteria, can grow under conditions that, at the same time, prevent the development of other microorganisms. As a result, since sterilization can be avoided, fermentation procedures can be more straightforward, and the presence of highly trained microbial fermentation engineers may be avoided.

Moreover, as these extremophile bacteria are more robust, they are also more stable under changing growth conditions, allowing the automation of the procedures (Chen and Jiang 2018). In future fermentation techniques, the problem of freshwater shortage could be solved by recycling the fermentation broth or using seawater rather than fresh water. The fermentation broth is rich in substances such as quorum-sensing molecules, proteins, polysaccharides, genetic materials, and lipids that could be used in cellular metabolic processes and thus become nutrients for the cells still (Yue et al. 2014). Another step in the prospects of fermentation processes will be favoring anaerobic or microaerobic microorganisms. Indeed, today’s fermentation processes use aerobic bacteria that need a lot of oxygen to grow and to convert substrates into products, especially in large-scale bioreactors, in which air compressors provide oxygen. However, since oxygen solubility in water is low, most of these molecules escape into the air, passing through the bioreactors. One solution adopted is the overexpression of bacterial hemoglobin to improve the oxygen uptake, but the use of anaerobic/microaerophilic bacteria would really help and would also minimize energy consumption, especially during product formation. Furthermore, using microorganisms growing in a wide range of temperatures will save even more energy (Ouyang et al. 2018).

Currently, glucose derived from hydrolysate starch is mainly used to produce several products. In future fermentation, waste substrates, such as molasses, activated sludge, cellulose, hydrolyzed sugars, methane, CO, and H_2_, should be used as nutrients for cellular growth, avoiding waste production. Future fermentation will rely on modifying and controlling bacterial cell morphology and self-aggregation cell ability to more simply separate cells and fermented broth only by gravity, thus without using centrifugation and filtration techniques. These two expensive and time-consuming methods are currently used to separate smaller cells and heavily fermented broth (Wang et al. 2019). Fermentation industries also create a lot of waste water composed mainly of organic cell debris and inorganic salts. Waste products could be treated to become nutritive molecules for the cell to grow again. In this way, the treated fermented broth could return to the bioreactors as food for cell growth and avoid wastewater generation (Tang et al. 2017). Moreover, new fermentation approaches will focus on using bacteria resistant to extreme conditions. For instance, it was reported that the bacterium *Halomonas campaniensis* sp. LS21 was able under non-sterilization conditions continuously for several days without contamination (Yue et al. 2014). Most of the current fermentation processes are run batch or fed-batch and require a lot of heavy manual controls (Chen and Jiang 2018).

Moreover, using plastic materials will reduce bioreactors’ weight and enhance structures’ transparency. Using cement instead will allow the building of larger bioreactors like building skyscrapers. Future fermentations will be conducted under sterilization conditions. The fact that future fermentations will be done under non-sterile and in continuous conditions controlled by artificial intelligence (AI) will reduce even more water and energy consumption (Chen and Liu 2021). All fermentation processes generate supernatants and solid biomass as wastes. Both these two products need to be treated. For this, future fermentations can be designed to produce small molecular extracellular products and insoluble intracellular inclusion bodies, i.e., polyhydroxyalkanoates (PHA), polyphosphates, and so on. In this way, there would be a more significant recovery of products, otherwise eliminated, starting from a single process, improving the process economy (Ma et al. 2020).

## Conclusion

The scientific investigations have provided deep insight into fermentation technology’s benefits regarding human health, food properties, and ecological well-being. Intriguingly, fermented functional foods have emerged as a critical subsector of the functional food industry, which has grown steadily over the past few years. Using various microbial species, particularly yeasts and various LAB strains, has significant industrial uses and even more promising future benefits in developing healthy cultured food and beverages. Fermentation has many potential health benefits, including producing beneficial lactic acid bacteria, ease of metabolism, increased nutritional availability, improved state of mind and behavior, and cardiovascular health benefits. Dairy and meat are the most popular fermented products widely consumed. On the other side, along with dairy and meat, fermented fruit and vegetables are well known for their worldwide consumption and health benefits. Increased awareness of alternative proteins has recently expanded fermented products with core ingredients of insects and seaweed. Moreover, to maximize the bioconversion efficiency of food waste, it is necessary to optimize the food-derived waste and use cutting-edge biotechnology methods. Innovative techniques like precision fermentation are well-suited to manufacture desired proteins, lipids, and carbohydrates because they enable the generation of molecules with similar constitutions as their conventionally farmed counterparts. In the future, fermentation industries will operate continuously, automation can be installed, and this will lead to a reduction in human involvement and the prevention of mistakes. In future fermentation, low-cost materials such as plastics, cement, or ceramic can be used instead of steel. In the bargain, Artificial Intelligence will be in charge of controlling fermentation, thus providing more efficient, sterile, and less energy, water, and workforce processes.

## Data Availability

The data that support the findings of this study are available from the corresponding author, [Shahida Anusha Siddiqui], upon reasonable request.
